# Distinct Immune Homeostasis Remodeling Patterns after HLA‐Matched and Haploidentical Transplantation

**DOI:** 10.1002/advs.202400544

**Published:** 2024-09-03

**Authors:** Huidong Guo, Liping Guo, Bixia Wang, Xinya Jiang, Zhigui Wu, Xiao‐Dong Mo, Yu‐Qian Sun, Yuan‐Yuan Zhang, Zhi‐Dong Wang, Jun Kong, Chen‐Hua Yan, Xiao‐Jun Huang

**Affiliations:** ^1^ National Clinical Research Center for Hematologic Disease Beijing Key Laboratory of Hematopoietic Stem Cell Transplantation Peking University People's Hospital Peking University Institute of Hematology Peking University Beijing 100044 China; ^2^ Research Unit of Key Technique for Diagnosis and Treatments of Hematologic Malignancies Chinese Academy of Medical Sciences Beijing 2019RU029 China; ^3^ Peking‐Tsinghua Center for Life Sciences Academy for Advanced Interdisciplinary Studies Peking University Beijing 100871 China

**Keywords:** allogeneic hematopoietic stem cell transplantation, immune homeostasis, immune reconstitution, immune tolerance, ZNF683

## Abstract

Allogeneic hematopoietic stem cell transplantation (allo‐HSCT) is a widely used treatment for a variety of hematopoietic disorders, and also provides a valuable platform for investigating the development of donor‐derived immune cells in recipients post‐HSCT. The immune system remodels from the donor to the recipient during allo‐HSCT. However, little is known about the cell profile alterations as donor homeostasis rebalances to recipient homeostasis following HSCT. Here, multi‐omics technology is applied at both the single cell and bulk sample levels, as well as spectrum flow cytometry and fluorescent transgenic mouse models, to dissect the dynamics of the rebalanced homeostatic immune system in recipients after allo‐HSCT. The data reveal that all immune subpopulations observed in donors are successfully restored in recipients, though with varying levels of abundance. The remodeling of immune homeostasis exhibits different patterns in HLA‐matched and haploidentical HSCT, highlighting distinct biases in T cell reconstitution from the central and peripheral pathways. Furthermore, *ZNF683* is critical for maintaining the persistence and quiescence of CD8 T‐cell in haploidentical HSCT. The research can serve as a foundation for developing novel strategies to induce immune tolerance.

## Graphical abstract

Using multi‐omics technology and fluorescent transgenic mouse models, the dynamics of the rebalanced homeostatic immune system are dissected in recipients after allo‐HSCT. The findings reveal that inhibiting peripheral reconstituted T cell activation, restoring thymus function, and promoting Tcell central pathway reconstitution are crucial for achieving immune homeostasis following haplo‐SCT. Furthermore, ZNF683 acts as a checkpoint in maintaining the quiescence of CD8 T cells.

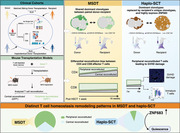

## 1 Introduction

The homeostatic immune system in a healthy state represents a dynamic balance between immunogenicity and immune tolerance.^[^ ^1^ ^]^ The immune system allows an appropriate immune response to eliminate foreign antigens while preventing immune overactivation to avoid tissue damage to the host. At steady‐state, a delicate equilibrium is maintained in healthy individuals through the orchestrated interplay of positive effectors, such as T cells and NK cells, alongside negative regulators, including Tregs and tolerogenic dendritic cells (DCs). This intricate balance ensures the maintenance of immune homeostasis. Allogeneic hematopoietic stem cell transplantation (allo‐HSCT) is not only a curative therapy for hematological malignancies,^[^ ^2^ ^]^ but has also been used to remodel the immune system to a homeostatic state, such as inducing allograft tolerance in solid organ transplantation and treating autoimmune diseases.^[^ ^3^ ^]^ In the context of allo‐HSCT, donor‐derived immune cells not only mount an active immune response against infections or leukemia cells but also establish tolerance toward the recipient, thereby preventing graft rejection or graft‐versus‐host disease (GVHD). Consequently, recipients can achieve a state where they no longer require immunosuppressive conditioning and maintain a harmonious immune response following HSCT. However, little is known about the responsible genes or relevant cell types that remodel immune homeostasis after allo‐HSCT. Furthermore, it remains unclear whether there are any disparities in immune cell distribution in the homeostatic state between healthy individuals and recipients following allo‐HSCT.

The human leukocyte antigen (HLA) complex plays a critical role in determining whether a person's immune system will accept tissue or cells from another individual without triggering an immune response.^[^ ^3b^ ^]^ Historically, HLA‐matched sibling donor transplantation (MSDT) was the only option for allo‐HSCT as allografts can provoke a strong immune response in the recipient, potentially leading to graft rejection and GVHD.^[^ ^4^ ^]^ However, recent advances in haploidentical stem cell transplantation (haplo‐SCT) have successfully controlled the alloimmune response, resulting in significantly improved outcomes.^[^ ^5^ ^]^ Although both MSDT and haplo‐SCT can effectively rebalance the donor‐derived immune system to homeostasis in recipient following allo‐HSCT, the changes that occur during the immune homeostasis restoration in the recipient remain poorly understood. Additionally, it is unclear whether MSDT and haplo‐SCT share similar immune homeostasis remodeling patterns during HSCT. Furthermore, there is limited knowledge regarding the cellular and molecular atlas of the donor‐derived immune system that crosses the HLA barrier and achieves homeostasis in recipients undergoing haplo‐SCT.

Innate immunity, including monocytes, granulocytes and natural killer (NK) cells, typically recovers quickly following HSCT; while adaptive immune cells, particularly T cells, tend to recover at a much slower pace and may take at least 1–2 years to fully restore.^[^ ^6^ ^]^ The reconstitution of the T cell compartment is crucial beyond their essential role in the defense against opportunistic pathogens, as they are also the key effectors of graft‐versus‐leukemia (GVL) immune responses. In T‐cell‐repleted HSCT setting, regeneration of the T cell compartment is accomplished by peripheral and central reconstitution pathways in parallel. The first peripheral pathway involves mature donor T cells undergoing lymphopenia‐induced homeostatic proliferation and alloactivation, leading to skewing of the T cell receptor (TCR), contraction of TCR repertoire, and alloreactivity that can cause GVHD. The second central pathway generates de novo naïve T cells from hematopoietic progenitors through a thymus‐dependent regenerative mechanism. This mechanism not only allows for the development of a new T cell pool with broad TCR diversity but also educates T cells to recognize the recipient as “self‐antigen”, leading to the establishment of tolerance toward the recipient.^[^ ^6^, ^7^ ^]^ However, the heterogeneity of allografts consisting of both donor mature T cells and hematopoietic progenitors, along with the fact that these cells rarely achieve perfect synchrony and exhibit nondeterministic dynamics, makes it challenging to track the reconstitution dynamics of the immune system, especially for T cells, in recipients. This leads to the dynamics of T cell regeneration from the peripheral and central pathway during the restoration of immune homeostasis after HSCT remain ambiguous.

In this study, we utilized the advantages of multi‐omics technology at both the single cell and bulk sample levels, as well as spectrum flow cytometry and fluorescent transgenic mouse models, to dissect the dynamics of the rebalanced homeostatic immune system in recipients after allo‐HSCT. We first delineated the cellular and molecular atlas of the immune system in recipients who successfully rebalanced immune homeostasis after allo‐HSCT, defined as recipients who had received allo‐HSCT for more than one year, with long‐lasting absence of GVHD, infection or relapse, and had stopped immunosuppressive regimen treatment. Furthermore, we established syngeneic (MHC‐matched) and haplo‐identical (MHC‐haplomatched) transplantation models using fluorescent transgenic mouse models, successfully visualizing the dynamics of T cell reconstitution through the peripheral and central pathway in different transplantation models. We found different patterns of immune homeostasis remodeling in HLA‐matched and HLA haplo‐matched transplantation models and characterized key effectors that contributes to crossing the HLA barrier and rebalancing immune homeostasis for haplo‐SCT. Our research provides novel strategies for inducing immune tolerance and new mechanistic insights into transplantation immunology.

## 2 Results

### 2.1 Global Analysis of Reconstituted Cell Subsets in Recipients after Allo‐HSCT

**Figure 1:**
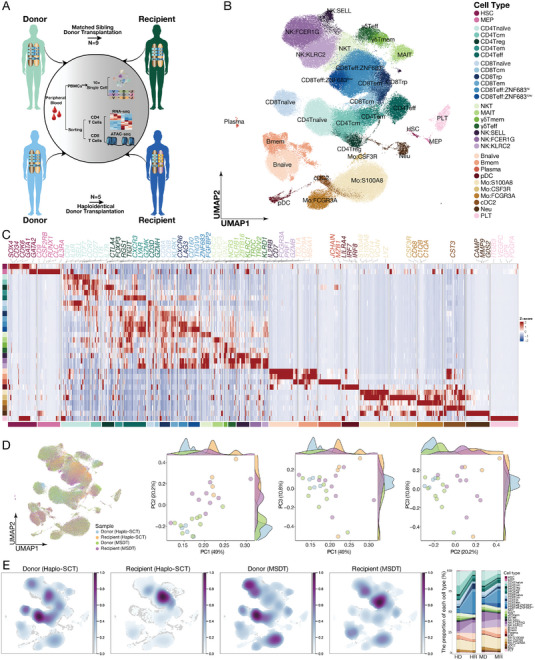
Transcriptome profiling in homeostatic donor‐recipient pairs after allo‐HSCT. A) Graphic overview of the experimental settings. PBMCs were collected from paired donors and recipients in the MSDT (N = 9) or haplo‐SCT (N = 5) group and processed for scRNA‐seq (twelve pairs of samples were subjected to scTCR‐seq at the same time), one donor‐recipient pair in the haplo‐SCT group were sorted CD8^+^ T cells and subjected to scRNA‐seq and scTCR‐seq. CD4^+^ and CD8^+^ T cells were sorted and subjected to RNA‐seq and ATAC‐seq. B) UMAP visualization of hematopoietic and immune cells from PBMCs, colored by cell type. HSC, hematopoietic stem cells; MEP, megakaryocyte and erythroid progenitor; MAIT, mucosal‐associated invariant T cells; γδT, T cells with TCR γ and δ chain. C) Heatmap of the mean expression value of the top 50 genes for each cell type and manually labeled marker genes. The scaled count values of genes are indicated by color intensity. See Table S3 (Supporting Information) for all marker genes. D) Distribution of hematopoietic and immune cells across all cell types and principal component analysis (PCA) of all samples based on the ratio of cell proportion, colored based on MSDT or haplo‐SCT donor and recipient. E) Cell density and average cell cluster frequency is shown as a fraction of total cells for donors and recipients from MSDT or haplo‐SCT group; HD, haplo‐SCT donor; HR, haplo‐SCT recipient; MD, MSDT donor; MR, MSDT recipient. See Tables S1 and S2 (Supporting Information).

We present an in‐depth cellular and molecular analysis of recipients who achieved immune system homeostasis after allo‐HSCT. The recipient who achieved immune homeostasis is defined as the recipient who underwent allo‐HSCT for more than one year, with a sustained absence of GVHD, infections, or relapse, and had stopped immunosuppressive regimen treatment. The paired donor for each recipient was also enrolled at the same time. We performed a single‐cell level analysis of transcriptome and TCR clonotype from 9 pairs of MSDT donor‐recipient pairs and 5 pairs of haplo‐SCT donor‐recipient pairs (**Figure** **1**A; Figure S1A and Tables S1 and S2, Supporting Information). In parallel, we sorted CD3^+^CD4^+^ T cells and CD3^+^CD8^+^ T cells and conducted RNA‐seq and ATAC‐seq analyses to ascertain the transcriptomic and epigenomic profiles (Figure 1A). After filtering, we retained 181973 cells for analysis, of which 126983 were derived from MSDT donor‐recipient pairs and 54990 from haplo‐SCT donor‐recipient pairs, with a mean of 1776 genes per cell (Figure 1B). Based on differential expression analysis and the top marker genes ranked by significance of standardized expression, we manually annotated 5 lineage populations, including the hematopoietic progenitor compartment, T/NK lineage cells, B lineage cells, myeloid lineage cells and platelets (Figure 1B,C). We further identified 30 subpopulations in the 5 lineages based on the expression of cell type‐associated genes (Figure 1B,C; Figure S1B,C and Table S3, Supporting Information).

In the hematopoietic progenitor compartment, hematopoietic stem cells (HSCs) are characterized by high expression of *CD34* and *SOX4*. Megakaryocyte and erythroid progenitor (MEP) showed high levels of *CLC* and *GATA2*. T cell compartment includes CD4 naïve T cells (CD4 Tnaïve), CD4 central memory T cells (CD4 Tcm), CD4 regulatory T cells (CD4 Treg), CD4 effector memory T cells (CD4 Tem), CD4 effector T cells (CD4 Teff), CD8 Tnaïve, CD8 Tcm, CD8 regulatory precursor T cells (CD8 Trp, which is we defined a distinct CD8 T cell subtype that exhibits a high expression level of HLA class II molecules such as *HLA‐DPA1* and *HLA‐DRB1*, accompanied with high expression level of inhibitory genes such as *TIGIT* and *LAG3*), CD8 Tem, CD8 effector T cells with highly expressed transcription factor (TF) *ZNF683* (CD8Teff:ZNF683^hi^) and CD8 effector T cells with low expression of *ZNF683* (CD8Teff:ZNF683^low^). We identified natural killer T cells (NKT) by a high expression level of cytotoxic genes such as *GZMB*, *NKG7* and *PRF1*, accompanied by the expression of TCR αβ chain. Mucosal‐associated invariant T cells (MAIT) were characterized by highly express *KLRB1* (also known as *CD161*), *CD8A* and *CXCR6* according to previous studies.^[^ ^8^ ^]^ We identified two γδ T cell clusters, which is memory γδ T cells with a high expression level of *IL7R* and *CD27* (γδTmem), and effector γδ T cells with a high expression level of cytotoxic genes such as *FGFBP2*, *NKG7* and *PRF1* (γδTeff). Based on previous studies of the single cell features of human NK subsets,^[^ ^9^ ^]^ we characterized three NK cell clusters. The first cluster resembles CD56^bright^ NK cells and highly expresses *SELL* (also known as *CD62L*) and the inflammatory cytokine *XCL2* (NK:SELL). The other two clusters are similar to CD56^dim^ NK cells, expressing *FCGR3A* (also known as *CD16*). One of these two clusters is highly expresses *FCER1G* (NK:FCER1G) and the other highly expresses *KLRC2* (NK:KLRC2). In the B lineage, we defined three clusters, including naïve B cells with high expression level of *TCL1A*, memory B cells shows high level of *MS4A1*, and plasma cells with high expression level of *MZB1* and *JCHAIN*. In the myeloid lineage, we characterized 3 types of clusters, including DCs, monocytes and neutrophils. According to previous studies of the features of human DC subsets,^[^ ^10^ ^]^ we identified plasmacytoid DCs (pDCs) with high expression level of *LILRA4*, *IRF7* and *IRF8*; type 2 classical DCs (cDC2s) with high level of *CD1C*. We have identified two clusters of classical monocytes and one cluster of nonclassical monocytes based on the expression level of *CD14*. Mo:S100A8 shows a high expression level of *CD14*, as well as *S100A8*, *LYZ*, *CST3* and *IFI30*; Mo:CSF3R exhibits a relatively lower level of *CD14* expression and expresses neutrophil signature genes such as *CSF3R* and *ZEB2*; Mo:FCGR3A is identified as nonclassical monocytes with a high expression level of *CD68* and *IFI30*. Additionally, we have identified a cluster of neutrophils that exhibit a high expression level of *S100A8*, *S100A9*, *FCGR3B* and *CSF3R*. All cell subsets were observed in both donors and recipients, with no significant variances detected within samples of each group (Figure 1D,E), suggesting successful immune reconstitution in recipients following allo‐HSCT.

### 2.2 Sub‐Clustering of T Cells Identifies a Distinct Population of CD8 Regulatory Cells

**Figure 2:**
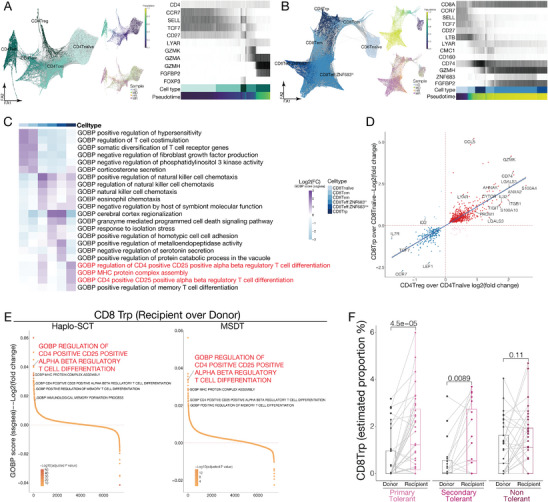
CD8 Trp cells contribute to the remodeling of immune homeostasis after allo‐HSCT. A) Trajectory analysis of CD4^+^ T cells, colored by subpopulations, pseudotime, and MSDT or haplo‐SCT donors and recipients. The heatmap shows the gene expression progression of CD4 T cell subsets along the pseudotime axis. Starting from the CD4 Tnaïve cells as the initial point, followed by CD4 Tcm, CD4 Treg, CD4 Tem, and concluding with CD4 Teff cells at the opposite end. B) Trajectory analysis of CD8^+^ T cells, colored by subpopulations, pseudotime, and MSDT or haplo‐SCT donors and recipients. The heatmap shows the gene expression progression of CD8 T cell subsets along the pseudotime axis. Starting from the CD8 Tnaïve cells as the initial point, followed by CD8 Tcm, CD8 Trp, CD8 Tem, and concluding with CD8 Teff:ZNF683^hi^ and CD8 Teff:ZNF683^low^ cells at the opposite end. C) Heatmap shows the top 5 differentially enriched GO terms for each CD8 T‐cell subpopulation. The enrichment level is indicated by color intensity. D) Gene enrichment in CD8 Trp cells and CD4 Treg cells relative to CD8 Tnaïve and CD4 Tnaïve cells, respectively. Key genes enriched in each group are labeled. E) Ranking of significantly differentially enriched GO terms among CD8 Trp cells in the indicated paired donor‐recipient groups. F) The estimated proportion of CD8 Trp by deconvolution analysis of public bulk RNA‐seq data. Primary tolerant patients and paired donors (N = 36); secondary tolerant patients and paired donors (N = 20); non‐tolerant patients and paired donors (N = 37). Paired *t‐*test.

In order to provide a more comprehensive assessment of T cell reconstitution during the remodeling of immune homeostasis after allo‐HSCT, we conducted separate analyses of the T cell clusters. Our trajectory analysis revealed that naïve T cells could develop into central memory and effector memory T cells, and subsequently into effector T cells in both CD4 and CD8 T cell subsets (**Figure** **2**A,B). This finding is consistent with the cell subset signature identified in our study. Consistent with previous results, all T cell subsets characterized in the donors were successfully reconstituted in their paired recipients (Figure 1D and Figure 2A,B).

We defined a distinct CD8 T cell subtype, CD8 regulatory precursor T cells (CD8 Trp), that exhibits a high expression level of HLA class II genes (*CD74*, *HLA‐DPA1* and *HLA‐DRB1*) and the inhibitory genes (*TIGIT* and *LAG3*) (Figures 1C and 2B; Figure S1B,C and Table S3, Supporting Information). Trajectory analysis revealed that CD8 Trp cells are situated close to CD8 Tcm cells and in front of CD8 Tem cells in the development trajectory, similar to the position of CD4 Treg cells (Figure 2A,B). These results indicated that CD8 Trp cells exhibit a precursor signature. GO analysis revealed that the CD4 Treg cell differentiation‐related genes were enriched in the CD8 Trp cluster (Figure 2C). Moreover, we found that the transcriptional profiling overlapped between CD4 Tregs and CD8 Trp when we separately compared the gene expression enrichment of CD4 Treg or CD8 Trp to CD4 Tnaive or CD8 Tnaive (Figure 2D). Genes enriched in both the CD8 Trp and CD4 Treg subpopulations included *TIGIT* and the TF *PRDM1* (Figure 2D), and PRDM1 was identified as essential for maintaining T‐cell self‐tolerance and homeostasis.^[^ ^11^ ^]^ These results indicated that CD8 Trp cells may have an immunoregulatory role. Several studies have shown that CD8^+^ T cells that highly express HLA‐DR, CD38, and inhibitory markers, such as PD‐1 and TIGIT, are active subsets and persist in patients with severe virus infection disease^[^ ^12^ ^]^ or patients who receive immune therapy.^[^ ^13^ ^]^ However, other studies have shown that CD8^+^HLA‐DR^+^ T cells suppress the immune response in vitro.^[^ ^14^ ^]^ Here, in our study, we found that CD8 Trp cells highly express HLA II‐related genes while expressing low levels of *CD38* (Figure S2A,B, Supporting Information). The proliferative abilities of CD8 Trp cells were not significantly different from those of other CD8 T subpopulations (Figure S2C, Supporting Information). These results indicated that CD38 might be an active marker of CD8 T cells and that HLA‐DR might correlate with the immunoregulatory function of CD8 T cells. Moreover, we found that the GO terms associated with CD4 Treg cell differentiation‐related genes and MHC protein complex assembly related genes were enhanced in the recipients compared to their paired donors from both haplo‐SCT and MSDT groups (Figure 2E). These results suggested that CD8 Trp cells may contribute to remodel immune homeostasis in recipients following HSCT. To validate this hypothesis, we performed deconvolution analysis on previously published bulk RNA‐seq datasets of paired donor‐recipient samples from MSDT. The deconvolution was carried out using the average transcriptome derived from the 30 cell types identified in our scRNA‐seq datasets. The published transcriptomes of PBMCs were derived from primary tolerant patients (patients who did not develop acute or chronic GVHD and whose immunosuppression drugs had been withdrawn since several months at the time of sample), secondary tolerant patients (patients who experienced acute or chronic GVHD but to whom immunosuppression drugs were lastly stopped) and non‐tolerant patients (patients who developed acute or chronic GVHD and to whom physicians were unable to stop immunosuppression drugs).^[^ ^15^ ^]^ The results showed that the proportion of CD8 Trp cluster was significantly increased in recipients compared to their paired donors from both the primary tolerant and secondary tolerant groups, while there is no significant difference of CD8 Trp cluster proportion between recipients and their paired donors in the non‐tolerant group (Figure 2F). These results indicated that CD8 Trp subpopulation may act as regulatory cells and contribute to remodeling immune homeostasis in recipients after allo‐HSCT.

### 2.3 Redistribution of T Cell Subsets in Recipients Restoring Immune Homeostasis after Haplo‐SCT and MSDT

**Figure 3:**
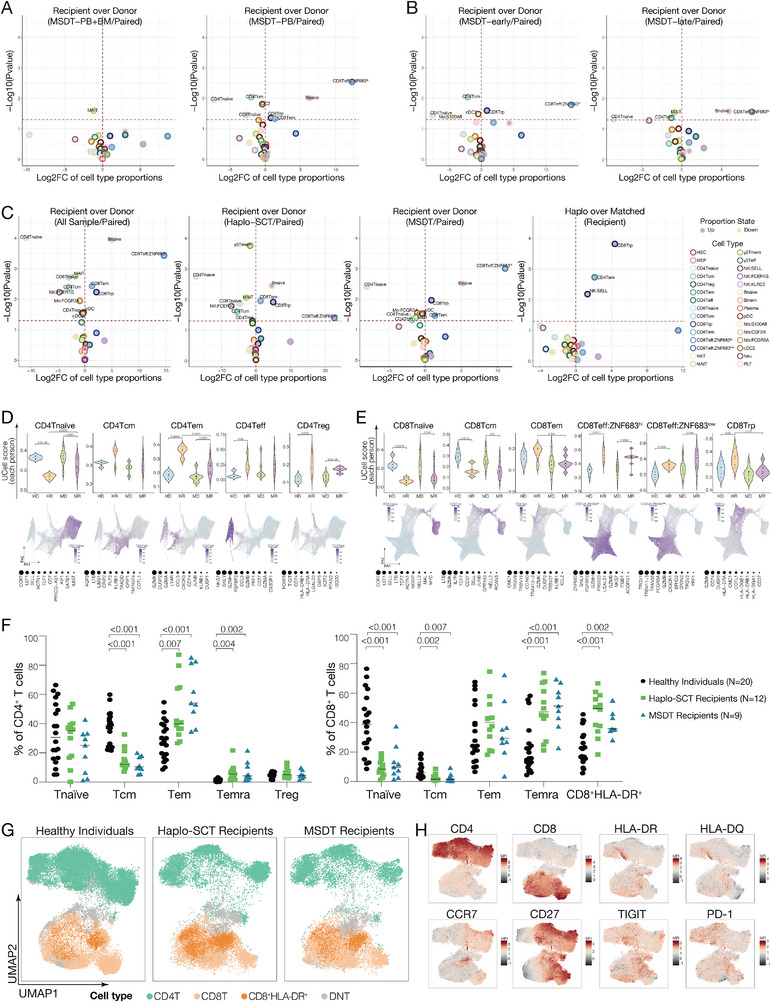
T‐cell subpopulations distribution in recipients with rebalanced homeostasis after allo‐HSCT. A) Quantification of cell cluster frequency among recipients and paired donors in the MSDT recipients received grafts composed of peripheral blood combined with bone marrow or only peripheral blood. B) Quantification of cell cluster frequency among recipients and paired donors in the MSDT recipients at the early‐stage (<2 years) or late‐stage (>2 years) post‐HSCT. C) Quantification of cell cluster frequency among recipients and paired donors in the haplo‐SCT or MSDT group. The increased and decreased cell types are shown in purple and light brown, respectively. The horizontal red dashes indicate the boundary of a P value equal to 0.05. Paired limma test were used for comparisons between donors and recipients. Unpaired limma test were used for comparisons between haplo‐SCT recipients and MSDT recipients. D‐E) UCell scores calculated for the top 10 marker genes in CD4 T (D) and CD8 T (E) cell subtypes. Each dot in the violin plot represents the average UCell score for each CD4 or CD8 T‐cell subtype in each individual. The PAGA plots showing the distribution of the UCell score in each cell. The top 10 genes used to calculate the UCell score program are ranked from 1 to 10, and the size of the dots indicates the rank of each gene within the program. HD, haplo‐SCT donor; HR, haplo‐SCT recipient; MD, MSDT donor; MR, MSDT recipient. F) T cell subsets reconstitution level in CD4^+^ T cells (left) or CD8^+^ T cells (right) in recipients who have achieved immune homeostasis after allo‐HSCT, detected by spectrum flow cytometry. One‐way‐ANOVA. G) UMAP visualization of spectrum flow cytometry detected CD3^+^ T cells from recipients who have achieved immune homeostasis after allo‐HSCT. H) Heatmap of the expression level of indicated markers in CD3^+^ T cells from recipients who have achieved immune homeostasis after allo‐HSCT.

To gain further insights into the factors that influence the immune reconstitution in recipients undergoing allo‐HSCT, we performed a comparative analysis of immune profile distribution in paired MSDT donor‐recipient under different HSCT conditions. It is showed that the immune profile distribution in recipients was more similar to that of their paired donors when recipients received grafts composed of peripheral blood combined with bone marrow (**Figure** **3**A). Moreover, we categorized recipients into two groups: an early‐stage comprising recipients who were less than 2 years post‐HSCT, and a late‐stage group consisting of those who were over 2 years post‐HSCT. The results showed that CD8 Teff:ZNF683^hi^ cells were expanded in both recipient groups when compared with their paired donors, and CD8 Trp cells were found to expand exclusively in recipients at the early‐stage after HSCT (Figure 3A).

We further compared the subpopulation distribution in different transplantation models. Although all immune cell subpopulations from donors were reconstituted in the paired recipients, the abundance of immune cell subpopulations showed disparities (Figure 3C; Tables S4 and S5, Supporting Information). The most remarkable changes in immune subpopulations between recipients and donors were observed in the T‐cell compartment (Figure 3C). Our analysis revealed three clusters of CD8^+^ T cells, including CD8 Teff:ZNF683^hi^, CD8 Tem, and CD8 Trp, as well as naïve B cells that were enriched in recipients from both haplo‐SCT and MSDT groups (Figure 3C; Table S5, Supporting Information). The expansion of these immune subpopulations may contribute to the restoration of immune homeostasis in recipients following allo‐HSCT. In contrast, CD4 Tnaïve cells were found to be enriched in the donors of both groups (Figure 3C; Table S5, Supporting Information). Although CD8 Trp cells were expanded in recipients from both haplo‐SCT and MSDT groups compared to their paired donors, this phenomenon was more pronounced in haplo‐SCT recipients when compared to MSDT recipients (Figure 3C; Table S5, Supporting Information). Moreover, although the frequency of CD4 Treg cells was decreased in haplo‐SCT recipients compared with their paired donors, the frequency of CD8 Trp cells was increased in contrast (Figure S2D, Supporting Information). The increased CD8 Trp subpopulations might act as regulatory cells and contribute to maintaining the tolerance of the donor‐derived immune system in recipients after haplo‐SCT. We further evaluated the function of T cell subpopulations by transcriptional program scores (UCell score). The results showed that CD4 Tnaive, CD8 Tnaive and CD8 Tcm transcriptional programs were downregulated and the effector T‐cell transcriptional program in CD4 Tem, CD8 Teff:ZNF683^hi^ and CD8 Teff:ZNF683^low^ were upregulated in recipients compared with their paired donors from both haplo‐SCT and MSDT groups (Figure 3D,E). Interestingly, although the quantity of CD4 Treg was decreased in haplo‐SCT recipients compared with their paired donors, the transcriptional program score was increased (Figure 3C,D). Taken together, these results demonstrated a redistribution of the T cell compartment in recipients following allo‐HSCT.

To validate our scRNA‐seq data, we enrolled additional 12 haplo‐SCT recipients and 9 MSDT recipients who have achieved immune homeostasis after allo‐HSCT and detected T cell subsets reconstitution by spectrum flow cytometry (Figure S3 and Table S6, Supporting Information). Consistent with our scRNA‐seq data, CD8 effector memory T cells re‐express CD45RA (termed TEMRA) and CD8^+^HLA‐DR^+^ T cells were increased in both haplo‐SCT and MSDT recipients compared with healthy individuals (Figure 3F,G). These CD8^+^HLA‐DR^+^ T cells also showed high expression levels of HLA‐II molecules (HLA‐DQ), memory cell signature markers (CCR7 and CD27), and inhibitory markers (TIGIT and PD‐1) at the protein level (Figure 3H). These results indicated that CD8^+^ T cells with a high expression level of HLA‐II molecules were expanded in recipients following allo‐HSCT and may contribute to the remodeling immune homeostasis in these recipients.

### 2.4 Differential TCR Clonotype Distributions of Effector T Cells in Haplo‐SCT Recipients and MSDT Recipients

**Figure 4:**
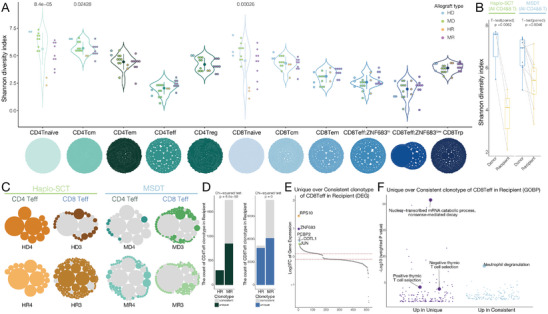
Differential T‐cell clone expansion patterns in haplo‐SCT recipients compared with MSDT recipients. A) TCR repertoire diversity in each T‐cell subpopulation. One‐way‐ANOVA test. B) TCR repertoire diversity index (Shannon) among recipients and paired donors in the haplo‐SCT or MSDT group. C) T‐cell clonotypes in CD4 Teff and CD8 Teff cells in the indicated paired donor‐recipient groups. Gray represents the consistent clonotype shared by paired donors and recipients, and color represents the unique clonotype possessed by the donors or recipients. D) The frequency of consistent or unique clonotypes in the indicated groups. E) The ranking of significantly DEGs in unique clonotype compared with consistent clonotype in CD8 Teff cells in recipients. F) GO enrichment analysis of specifically expressed genes in unique clonotype and consistent clonotype of CD8 Teff cells in recipients. HD, haplo‐SCT donor; HR, haplo‐SCT recipient; MD, MSDT donor; MR, MSDT recipient.

To investigate the dynamic changes in TCR clonotype after allo‐HSCT, we integrate analyzed single‐cell transcriptomes and TCR sequences. TCR diversity was decreased along the development trajectory from naïve T cells to effector T cells in both CD4 and CD8 T cells (**Figure** **4**A). TCR diversity was decreased in recipients compared to their paired donors from both haplo‐SCT and MSDT groups (Figure 4B). Interestingly, we found the abundance of each TCR clonotype in the paired donor‐recipient effector T cells differed between the haplo‐SCT and MSDT groups (Figure 4C; Figure S4, Supporting Information). To better decipher the alteration of their abundance, we defined a TCR clonotype shared by paired donors and recipients as a consistent TCR clonotype and vice versa as a unique clonotype. To focus on the analysis of TCR clonotype, we merged the CD8 Teff:ZNF683^hi^ cluster and CD8 Teff:ZNF683^low^ cluster. Specifically, for the CD8 Teff and CD4 Teff cells, the consistent TCR clonotypes remained the predominant TCR clonotype for the MSDT recipients, whereas the predominant clonotypes for the haplo‐SCT recipients were replaced by their unique clonotypes (Figure 4C,D; Figure S4, Supporting Information). TF *ZNF683* and *JUN* were highly expressed in unique clonotypes compared with consistent clonotypes in CD8 Teff cells (Figure 4E). Strikingly, genes related to the negative thymic T‐cell selection and positive thymic T‐cell selection pathway were enriched in unique clonotypes compared with consistent clonotypes in CD8 Teff cells (Figure 4F). These results indicated that these unique clonotype T cells underwent thymic development and reconstituted through the central pathway. Taken together, our analysis revealed the differential clonotype distributions in haplo‐SCT recipients and MSDT recipients, which may represent differential T cell reconstitution pathways following allo‐HSCT. This raises the question: Is there a differential bias in the reconstitution of T cells through the peripheral and central pathways between haplo‐SCT and MSDT?

### 2.5 The Dynamics of T Cell Reconstitution through the Peripheral and Central Pathway after HSCT In Vivo

**Figure 5:**
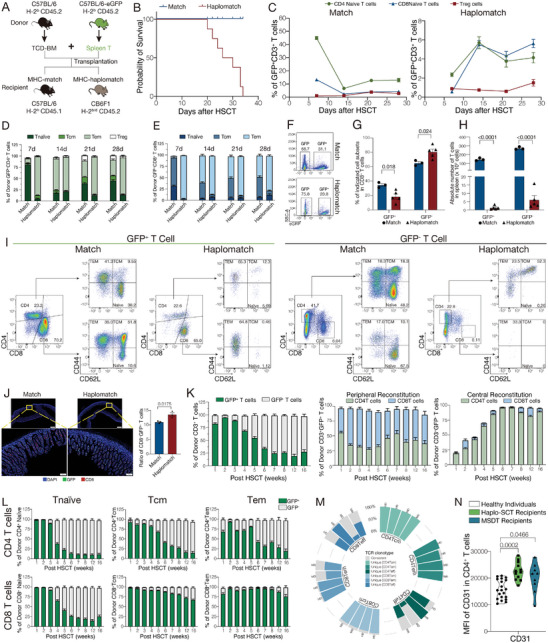
Dynamics of T cell reconstitution from the peripheral and central pathways in MHC matched and haplomatched transplantation models. A) Schematic illustration of the experimental design. T‐cell‐depleted bone marrow cells (TCD‐BM) from C57BL/6 mice (CD45.2, H‐2^b^) combined with spleen T cells from eGFP reporter mice (C57BL/6 background) at a 1:1 ratio was transplanted into lethally irradiated recipient mice. For the MHC‐match group, the recipient mice were C57BL/6 mice (CD45.1, H‐2^b^); for the MHC‐haplomatch group, the recipient mice were CB6F1 mice (CD45.2, H‐2^b/d^). B) Survival curves of MHC‐match and MHC‐haplomatch recipient mice. 8 mice per group. C) Proportion dynamics of indicated T cell subsets in peripheral blood from the MHC‐match and MHC‐haplomatch recipient mice after transplantation. D,E) T cell subsets in CD4 (D) and CD8 T cells (E) that reconstituted from the peripheral pathway in peripheral blood of MHC‐match and MHC‐haplomatch recipient mice after transplantation. F) Representative flow cytometry graph of donor‐derived T cells that reconstitute from the peripheral (GFP^+^) and central (GFP^−^) pathway in spleen of MHC‐match and MHC‐haplomatch recipient mice four weeks after transplantation. G,H) The ratio (G) and absolute number (H) of donor‐derived T cells that reconstitute from the peripheral (GFP^+^) and central (GFP^−^) pathway in spleen of MHC‐match and MHC‐haplomatch recipient mice four weeks after transplantation. I) Representative flow cytometry graph of donor‐derived T cell subsets that reconstitute from the peripheral (GFP^+^) and central (GFP^−^) pathway in spleen of MHC‐match and MHC‐haplomatch recipient mice four weeks after transplantation. J) T cell infiltration in small intestine of MHC‐match and MHC‐haplomatch recipient mice four weeks after transplantation. 3 mice per group. K) Reconstitution dynamics of T cells from the peripheral (GFP^+^) and central (GFP^−^) pathway in peripheral blood of MHC‐match recipient mice after transplantation. L) Reconstitution dynamics of T cell subsets from the peripheral (GFP^+^) and central (GFP^−^) pathway in peripheral blood of MHC‐match recipient mice after transplantation. M) Clonotype of indicated T cells in paired donor‐recipient from haplo‐SCT and MSDT groups. HD, haplo‐SCT donor; HR, haplo‐SCT recipient; MD, MSDT donor; MR, MSDT recipient. N) Median fluorescence intensity (MFI) of CD31 in CD4 T cells from healthy individuals (N = 20), haplo‐SCT recipients (N = 12), and MSDT recipients (N = 9). Unpaired *t*‐test for comparison between two groups. One‐way‐ANOVA for comparison in three groups. At least 5 mice per group in the assessment of immune reconstitution in peripheral blood. 3 mice in MHC‐match group and 5 mice in MHC‐haplomatch group in the assessment of immune reconstitution in spleen. Combined data from two independent experiments. The symbols represent individual mice. Error bars represent the mean ± SEM.

To visualize the dynamics of T cell reconstitution through peripheral and central pathway after HSCT in vivo, we employed fluorescent transgenic mice to establish transplantation mouse models. We transplanted T‐cell‐depleted bone marrow cells from C57BL/6 mice (CD45.2, H‐2^b^) combined with spleen T cells from eGFP reporter mice (C57BL/6 background) at a 1:1 ratio into lethally irradiated recipient mice. For the MHC‐matched group, the recipient mice were C57BL/6 mice (CD45.1, H‐2^b^); for the MHC‐haplomatch group, the recipient mice were CB6F1 mice (CD45.2, H‐2^b/d^) (**Figure** **5**A). This allowed us to distinguish the reconstitution pathway of donor‐derived T cells in recipient mice. GFP^+^ T cells represented T cells that reconstituted from the peripheral pathway, while GFP^−^ T cells represented T cells that reconstituted from the central pathway. The MHC‐haplomatch mice died from GVHD within 40 days after transplantation (Figure 5B; Figure S5A,B, Supporting Information). Thus, we were only able to observe T cell reconstitution for 4 weeks after transplantation in the MHC‐haplomatch groups. First, we detected the dynamics of T cell reconstitution in the peripheral blood by spectrum flow cytometry. We found GFP^−^ T cells derived from the central pathway expanded until 4 weeks after HSCT (Figure S5C, Supporting Information). In GFP^+^ T cells that reconstituted from the peripheral pathway, CD8^+^ T cells were more easily expanded than CD4^+^ T cells in both the MHC‐match and MHC‐haplomatch groups (Figure S5D, Supporting Information). This result is consistent with the fact that there is a transition in the CD4/CD8 ratio in recipients following allo‐HSCT. Due to thymic damage from irradiation, GFP^−^ T cells that reconstituted from the central pathway recovered until 4 weeks after transplantation in the MHC‐matched group, evidenced by the decreased ratio of immature CD4 and CD8 double negative T cells and double positive T cells found in GFP^−^ T cells from the peripheral blood (Figure S5E, Supporting Information). Importantly, we found that CD4 and CD8 naïve T cells reconstituted from the peripheral pathway underwent expansion in the MHC‐haplomatch groups compared with the MHC‐match groups two weeks after transplantation (Figure 5C). This led to the occurrence of GVHD, which further damaged the thymus function in the MHC‐haplomatch group (Figure S5A,E, Supporting Information). T cell subpopulations that reconstituted from the peripheral pathway also exhibited different expansion patterns in the MHC‐match and MHC‐haplomatch groups. In the CD4^+^ T cell compartment, naïve T cells underwent homeostatic expansion and remained at approximately 40% in the MHC‐match groups four weeks after transplantation, whereas in the MHC‐haplomatch groups, approximately 90% of CD4^+^GFP^+^ T cells were effector memory T cells at 4 weeks after transplantation (Figure 5D; Figure S5F, Supporting Information). In the CD8^+^ T cell compartment, there were mainly effector memory and central memory T cells in the MHC‐match groups while mainly effector memory T cells in the MHC‐haplomatch groups after transplantation (Figure 5E; Figure S5G, Supporting Information).

We further detected the dynamics of T cell reconstitution in the spleen at 4 weeks after transplantation. Although the ratio of T cells that reconstituted from the central pathway was higher in the MHC‐haplomatch group compared with the MHC‐match group, the absolute number of T cells from the central pathway was much lower in the MHC‐haplomatch group due to the thymus damage caused by GVHD (Figure 5F–H). Consistent with the results from peripheral blood, there were a large portion of unmature T cells in GFP^−^ T cell compartment in the spleen, and this was more pronounced in the MHC‐haplomatch group (Figure 5I). There were very few naïve CD4 and CD8 T cells in the GFP^−^ T cell compartment from the MHC‐haplomatch group (Figure 5I). Additionally, we observed that CD4 naïve T cells could reconstitute from both the peripheral and central pathway, whereas CD8 naïve T cells were predominantly derived from the central pathway in the MHC‐match group (Figure 5I; Figure S5H, Supporting Information). CD8 Tem cells that reconstituted from the peripheral pathway were also expanded in the spleen of the MHC‐haplomatch groups, consistent with the results from peripheral blood (Figure 5E,I). These results indicated that the expansion of the CD8 effector T cells from the peripheral pathway may contribute to the damage of GVHD in the haplo‐SCT groups. Therefore, we detected T cell infiltration in GVHD target tissues, including the small intestine and lung. We found a significant increase in the infiltration of CD8^+^GFP^+^ T cells in the small intestine and lung of the MHC‐haplomatch group compared with the MHC‐match group (Figure 5J; Figure S5I, Supporting Information). Taken together, these results demonstrated that it is crucial to inhibit peripheral reconstituted CD8 T cell expansion and promote thymus‐dependent central reconstitution for the restoration of immune homeostasis in haplo‐SCT.

Without the interference of GVHD, we were able to fully observe the dynamics of T cell reconstitution from the peripheral and central pathway after transplantation in the MHC‐match group. The ratio of GFP^−^ T cells that reconstituted from the central pathway was increased, and the ratio of GFP^+^ T cells that reconstitute from the peripheral pathway was decreased over time after transplantation (Figure 5K). The distribution of GFP^+^ and GFP^−^ T cells tended to stabilize in peripheral blood in MHC‐match mice at 7 weeks after transplantation (Figure 5K). T cell reconstitution from the central pathway in the spleen was fully restored at the 8 weeks after transplantation, as evidenced by the fact that most CD4 and CD8 T cells from the central pathway were mature T cells (Figure S5J‐L, Supporting Information). These results are consistent with previous studies indicating that damaged thymus function caused by irradiation can be recovered at 8 weeks after transplantation.^[^ ^16^ ^]^ We observed a persistence of approximately 25% GFP^+^ T cells up to 4 months after transplantation (Figure 5K). In the GFP^+^ T cell compartment, CD4 and CD8 T cells were present in a ratio of 1:1, whereas CD4 T cells were the majority in the GFP^−^ T cell compartment (Figure 5K). These results indicated that CD4 T cells could reconstitute from both the peripheral and central pathway, whereas CD8 T cells preferentially reconstitute from the peripheral pathway. We further analyzed the bias of reconstitution pathway in T cell subpopulations after transplantation. The results showed that both CD4 and CD8 naïve T cells were predominantly derived from the central pathway, and CD4 Tcm and Tem cells were predominantly derived from the central pathway, whereas CD8 Tcm and Tem cells were predominantly derived from the peripheral pathway (Figure 5L). These results indicated that T cell subsets from the CD4 and CD8 T cell compartments exhibited different biases in reconstitution from the peripheral and central pathways after transplantation.

Based on our observation of recipient‐unique clonotypes with high expression levels of thymus development‐related genes in our clinical scRNA‐seq data, we hypothesize that these unique clonotype T cells may have been derived from the central pathway, while the donor‐recipient consistent clonotype T cells may have been derived from the peripheral pathway. To validate this hypothesis, we analyzed the unique and consistent clonotypes in T cell subpopulations from paired donors and recipients of haplo‐SCT and MSDT. In the MSDT groups, both CD4 and CD8 Teff cells had a large proportion of consistent clonotypes in the recipients; In CD8 Tem cells there were nearly 50% consistent clonotypes, whereas most CD4 Tem cells were unique clonotypes in the recipients; In CD8 Tcm cells, we still observed consistent clonotypes persisting in the recipients, while almost all CD4 Tcm cells were unique clonotypes in recipients (Figure 5M). These results are consistent with the peripheral and central reconstitution patterns of CD4 and CD8 T cell subpopulations observed in the MHC‐match transplantation mouse model (Figure 5L). This confirms that donor‐recipient consistent clonotypes may be derived from the peripheral pathway, while recipient‐unique clonotypes may be derived from the central pathway. The majority of clonotypes in Teff, Tem and Tcm cells from both CD4 and CD8 T cells in haplo‐SCT recipients were recipient‐unique clonotypes (Figure 5M). Furthermore, spectrum flow cytometry analysis revealed that CD31, a marker of recent thymic emigrate (RTE) cells, was highly expressed in CD4^+^ T cells from haplo‐SCT recipients compared with health individuals (Figure 5N; Figure S5M, Supporting Information). These results indicated that these recipients successfully restored thymus function and effectively reconstituted T cells from the central pathway following haplo‐HSCT. Taken together, our data suggested that the restoration of thymus function and successful reconstitution of T cells from the central pathway may be key factors in remodeling recipient immune homeostasis after haplo‐SCT.

### 2.6 ATG did not Impact the T Cell Reconstitution Pathway during the Restoration of Immune Homeostasis after HSCT

ATG is an important clinical conditioning regimen in allo‐HSCT, and it has been shown to deplete donor‐derived CD8 T cells and human thymocytes in the thymic grafts in transplantation models.^[^ ^17^ ^]^ To decipher whether ATG contributes to the different T cell remodeling patterns observed in haplo‐SCT and MSDT, we compared the scRNA‐seq data between MSDT recipients with ATG conditioning and those without ATG conditioning. The results showed that there was no difference in the proportion of T cell subpopulations between MSDT recipients with and without ATG conditioning compared to their paired donors, except for CD8 Trp cells were increased in MSDT recipients with ATG conditioning compared to their paired donors, and CD8 Teff:ZNF683^hi^ cells were increased in MSDT recipients without ATG conditioning compared to their paired donors (Figure S5N, Supporting Information). The clonotype analysis also showed that ATG had little effect on the T cell clonotype distribution of the recipients following HSCT. There was no significant difference in the distribution of CD4 Teff, CD8 Teff and CD8 Tem consistent and unique clonotypes in recipients, regardless of whether they received ATG conditioning or not (Figures S4 and S5O, Supporting Information). These findings indicated that the administration of ATG had no discernible impact on T cell reconstitution from the peripheral and central pathways during the process of restoring immune homeostasis after HSCT. Taken together, these results suggest that ATG may affect T cell reconstitution at the early stage after HSCT but has limited effect on the distribution of immune subpopulations in recipients who have already restored immune homeostasis after HSCT.

### 2.7 *ZNF683* was Specifically Activated in CD8^+^ T Cells from Recipients in the Haplo‐SCT Group

**Figure 6:**
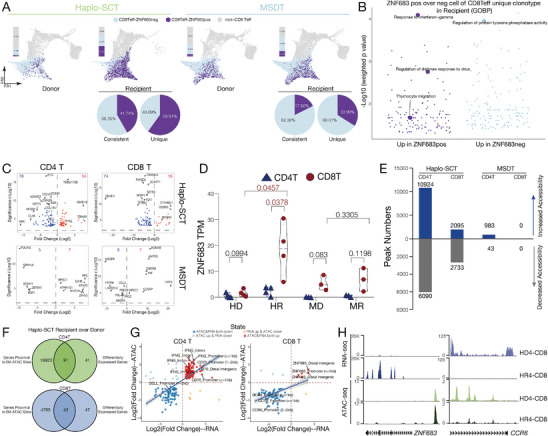
*ZNF683* is specific upregulated in CD8 T cells from haplo‐SCT recipients. (A) The distribution of ZNF683‐positive (more than one read) cells in the indicated groups visualized with PAGA plot. The pie chart shows the distribution of ZNF683‐positive cells and ZNF683‐negative cells in the CD8 Teffs of different clonotypes in the recipient. B) GO enrichment analysis of DEGs in ZNF683‐positive and ZNF683‐negative CD8 Teff cells with unique clonotype in recipients. C) Volcano plots showing DEGs of bulk RNAseq data in CD4 and CD8 T cells in the indicated paired donor‐recipient groups. D) The expression level of *ZNF683* in CD4 and CD8 T cells of paired donor‐recipient from haplo‐SCT or MSDT group. HD, haplo‐SCT donor; HR, haplo‐SCT recipient; MD, MSDT donor; MR, MSDT recipient. Two‐tailed paired t‐test. E) Overall open chromatin region (OCR) peak changes in CD4 and CD8 T cells in the indicated paired donor‐recipient groups. F) The overlap between DEGs and different accessibility‐proximal genes in CD4 and CD8 T cells from haplo‐SCT recipients compared with their paired donors. G) Integrated analysis of RNA‐seq and ATAC‐seq data. DEGs and DA peak‐proximal genes in CD4 and CD8 T cells from haplo‐SCT recipients compared with their paired donors are indicated. H) WashU browser views shows the gene expression level (RNA‐seq) and chromatin accessibility (ATAC‐seq) of specific genes in the indicated groups. HD, haplo‐SCT donor; HR, haplo‐SCT recipient; MD, MSDT donor; MR, MSDT recipient.

Our previous findings indicated that haplo‐SCT recipients achieved immune homeostasis with restored thymus function and T cell predominantly reconstituted through the central pathway. These T cells were characterized by high expression of the TF *ZNF683*, which is also known as HOBIT and is a homolog of PRDM1^[^ ^18^ ^]^ (Figure 4E). In further analysis, we found that ZNF683 positive CD8 Teff cells were specifically expanded in haplo‐SCT recipients, and over 50% of unique clonotype CD8 Teff cells from haplo‐SCT recipients were ZNF683 positive (**Figure** **6**A). Genes related to response to IFN‐γ, regulation of defense response to virus and thymocyte migration pathways were enriched in ZNF683pos cells (Figure 6B). Previous studies showed that *ZNF683* was highly expressed in patients after human cytomegalovirus (hCMV) infection,^[^ ^19^ ^]^ and there are 2 of 5 haplo‐SCT recipients infected with hCMV after HSCT (Figure S1A, Supporting Information). Therefore, we further validated the expression level of *ZNF683* in CD8 T cells from recipients who have rebalanced to immune homeostasis after allo‐HSCT by qPCR (Table S6, Supporting Information). The results showed that there is no difference of *ZNF683* expression level in CD8 T cells from haplo‐SCT recipients and MSDT recipients compared with healthy individuals (Figure S6A, Supporting Information). Interestingly, when we excluded recipients with hCMV infection after allo‐HSCT, the expression level of *ZNF683* was significantly increased in haplo‐SCT recipients compared with healthy individuals, whereas there was no difference of *ZNF683* expression level in MSDT recipients compared with healthy individuals (Figure S6B and Table S6, Supporting Information). Although there was no statistical significance, the expression level of *ZNF683* showed higher level in recipients without hCMV infection compared to recipients with hCMV infection from haplo‐SCT group, and the expression level of *ZNF683* showed lower level in recipients without hCMV infection compared to recipients with hCMV infection from MSDT group (Figure S6C, Supporting Information). These results indicated that the specific increased expression level of *ZNF683* in CD8 T cells from haplo‐SCT recipients is hCMV infection independent and might corelate with T cell reconstituted from the central pathway.

In parallel, we performed RNA‐seq and ATAC‐seq to analyze the transcriptome and epigenome dynamics in CD4^+^ T cells and CD8^+^ T cells after haplo‐SCT or MSDT (Figure 1A). Transcriptome analysis in bulk T cells showed that MSDT recipients shared a transcriptome profile similar to that of their paired donors, while haplo‐SCT recipients showed a disparate transcriptome profile compared with that of their paired donors (Figure 6C). *ZNF683* was specifically upregulated in CD8^+^ T cells from haplo‐SCT recipients compared with donors (Figure 6D). ATAC‐seq analysis also showed that the chromatin accessibility in MSDT recipients was similar to that in their paired donors, and the chromatin accessibility in haplo‐SCT recipients showed large disparity compared with that in their paired donors (Figure 6E). We further investigated the signature of differentially accessible (DA) regions in T cells from haplo‐SCT recipients compared with their paired donors. We found that 43.51% and 26.16% of DA regions were enriched in promoter regions in CD4^+^ T cells and CD8^+^ T cells, respectively (Figure S6D–I, Supporting Information). Increased levels of chromatin accessibility‐proximal genes, including *ZNF683*, were observed in CD4^+^ T cells from haplo‐SCT recipients compared with their paired donors (Figure S6J, Supporting Information). Motif enrichment in DA regions showed that TF FLI1 was activated and CTCF was repressed in CD4^+^ T cells, RUNX1 was activated and ERG was repressed in CD8^+^ T cells from haplo‐SCT recipients compared with their paired donors (Figure S6K, Supporting Information). Integrated analysis of transcriptome and epigenome profiles showed 91 genes in CD4^+^ T cells and 43 genes in CD8^+^ T cells were differentially expressed along with differential chromatin accessibility from haplo‐SCT recipients compared with their paired donors (Figure 6F). Notably, *ZNF683* was the most increased expression level gene and became accessible in CD8^+^ T cells from haplo‐SCT recipients compared with their paired donors (Figure 6G,H). These results indicated that TF ZNF683 was specifically activated in CD8^+^ T cells from haplo‐SCT recipients compared with their paired donors.

### 2.8 A High Level of *ZNF683* Promoted CD8 Effector T Cells into a Long‐Lived Quiescent State

**Figure 7:**
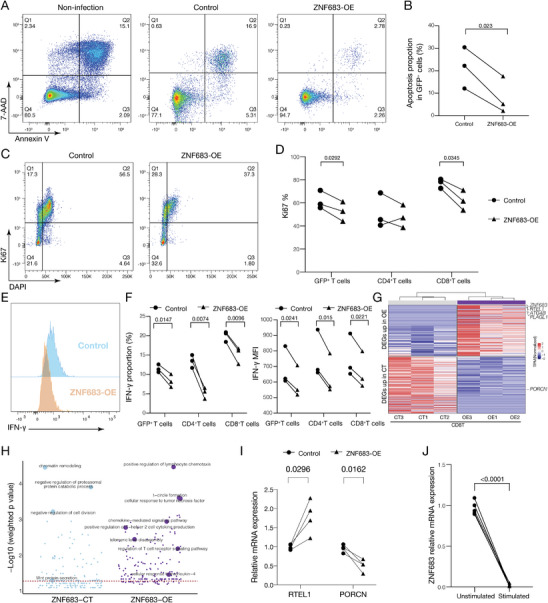
Human primary T cells highly expressing *ZNF683* exhibited a long‐lived quiescent state. A) Representative flow cytometry results showing apoptotic T cells in the uninfected, control, and ZNF683‐overexpressing groups. B) Comparison of the percentages of apoptotic T cells in the control and ZNF683‐overexpressing groups. C) Flow cytometric analysis of the proliferation of GFP^+^ cells. D) Percentage of Ki67^+^ cells among GFP^+^, GFP^+^CD4^+^ cells and GFP^+^CD8^+^ cells. E) IFN‐γ expression levels in GFP^+^CD8^+^ T cells. F) Left: Percentage of IFN‐γ^+^ cells among GFP^+^, GFP^+^CD4^+^ cells, and GFP^+^CD8^+^ cells; Right: Median fluorescence intensity (MFI) of IFN‐γ among GFP^+^, GFP^+^CD4^+^ cells, and GFP^+^CD8^+^ cells. G) Heatmap showing DEGs in CD8 T cells from the ZNF683‐overexpressing group compared with the control group. H) GO enrichment analysis of the DEGs of ZNF683‐overexpressing CD8^+^ T cells compared with control CD8^+^ T cells. I) *RTEL1* and *PORCN* expression level in ZNF683‐overexpressing CD8^+^ T cells compared with control CD8^+^ T cells. (N = 4) J) *ZNF683* expression level in CD8^+^ T cells from haplo‐SCT recipients (N = 6) who have rebalanced immune homeostasis, stimulated or unstimulated with CD3/CD28 microbeads. The experiments were replicated 3 times. Two‐tailed paired *t*‐test.

To explore the regulatory function of ZNF683 in human primary T cells, we overexpressed *ZNF683* in CD3^+^ T cells from the peripheral blood of healthy donors with control GFP‐expressing lentivirus (Control) or ZNF683‐overexpressing lentivirus (ZNF683‐OE) (Figure S7A,B, Supporting Information). The apoptosis level was decreased in ZNF683‐overexpressing T cells compared with that in control T cells (**Figure** **7**A,B). Proliferation was decreased in ZNF683‐overexpressing T cells compared with control T cells, and more ZNF683‐overexpressing CD8^+^ T cells were arrested in the G0 phase (Figure 7C,D; Figure S7C–E, Supporting Information). The secretion levels of IFN‐γ were decreased in ZNF683‐overexpressing T cells compared with that in control T cells (Figure 7E,F). These results indicate that a high expression level of *ZNF683* in CD8 T cells may promote T cells to enter a quiescent state. To further investigate the regulatory mechanism, we performed RNA‐seq on ZNF683‐overexpressing human primary CD8^+^ T cells. The results showed that *RTEL1*, which is critical for the stability, protection and elongation of telomeres,^[^ ^20^ ^]^ was upregulated in the ZNF683‐OE group compared with the control group (Figure 7G). Consistently, genes related to t‐circle formations and telomeric loop disassembly, which are involved in the telomere maintenance process allowing efficient telomere replication, were enriched in ZNF683‐overexpressing CD8 T cells compared with control T cells (Figure 7H). Moreover, we found that *PORCN*, which is an O‐acyltransferase regulating the activation of Wnt proteins,^[^ ^21^ ^]^ was downregulating in ZNF683‐overexpressing CD8 T cells compared with control T cells (Figure 7G). GO analysis also showed that the Wnt signaling pathway was downregulated in ZNF683‐overexpressing CD8 T cells compared with control T cells (Figure 7H). Consistently, we found that *LAG3* was positively correlated with *ZNF683* (Figure S7F, Supporting Information), and *ZNF683* positively corelated with genes enriched in negative regulation of cell cycle pathway and *ZNF683* negatively correlated with genes enriched in the Wnt signaling pathway (Figure S7G, Supporting Information). Furthermore, we validated the RNA‐seq results using qPCR. The results showed that in ZNF683‐overexpressing CD8 T cells, the expression level of *RTEL1* was increased while *PORCN* expression decreased compared to control T cells (Figure 7I). Taken together, ZNF683 might maintain T‐cell persistence by upregulating *RTEL1* and elongating T‐cell telomeres, while promoting T‐cell quiescence by downregulating *PORCN* and inhibiting the activation of the Wnt pathway.

Interestingly, we enriched CD8^+^ T cells from haplo‐SCT recipients who have rebalanced immune homeostasis and stimulated them with CD3/CD28 beads in vitro. *ZNF683* expression levels were strongly downregulated after stimulation (Figure 7J). Moreover, the expression level of *PORCN* was increased in CD8^+^ T cells after CD3/CD28 beads stimulation, indicating activation of the Wnt signaling pathway in activated CD8^+^ T cells (Figure S7H, Supporting Information). Taken together, these results indicated that a high expression level of *ZNF683* maintained CD8 T‐cell persistence and quiescence.

## 3 Conclusion

In this study, we present a detailed view of the cellular and molecular characteristics of donor‐derived immune cell remodeling homeostasis in recipients at the single‐cell level for the first time. All immune subpopulations characterized in donors were successfully reconstituted in recipients with varying levels of abundances, which indicated a redistribution of immune system compositions. A new CD8T regulatory cluster (CD8 Trp) was expanded in recipients and contributed to the remodeling of immune homeostasis in recipients following allo‐HSCT. Notably, we identify distinct remodeling patterns of immune homeostasis in MSDT and haplo‐SCT. In MSDT, the dominant clonotypes of donors tended to persist in recipients, while more unique clonotypes with a high expression level of thymus development related genes emerged in recipients than in their paired donors in the haplo‐SCT recipients. Subsequent transplantation mouse models validated that unique clonotypes represent T cells that reconstituted from the thymus‐dependent central pathway, while consistent clonotypes represent T cells that reconstituted from the peripheral pathway. These findings strongly suggest that the recovery of thymus function and the promotion of T cell central pathway reconstitution are crucial factors in the remodeling of immune homeostasis in recipients following haplo‐SCT. In recipients of haplo‐SCT with successful thymic regeneration, CD8 T cells reconstituted from the central pathway and exhibited a high expression level of the TF *ZNF683*. Further studies demonstrated ZNF683 as a checkpoint for maintaining CD8 T‐cell persistence and quiescence. ZNF683 is identified in NKT cells and tissue resident CD8 T cells (Trm) in mouse models,^[^ ^18^, ^22^ ^]^ it does not prominently identify human Trm cells.^[^ ^23^ ^]^ *ZNF683* is mainly expressed in circulating effector‐like T cells and correlates with T‐cell cytotoxic function in humans.^[^ ^19^, ^24^ ^]^ Our studies suggest a high level of *ZNF683* promoted the inactivation of CD8^+^ T cells, maintained CD8^+^ T‐cell replicative capacity and inhibited CD8^+^ T‐cell apoptosis, which is consistent with previous studies reporting that ZNF683 correlates with T‐cell persistence in humans.^[^ ^19^, ^25^ ^]^ Mechanismly, the high expression level of *ZNF683* might maintain T‐cell persistence by upregulating *RTEL1* and elongating T‐cell telomeres, while promoting T‐cell quiescence by downregulating *PORCN* and inhibiting the activation of the Wnt pathway. Further investigation is needed to explore how ZNF683 regulates the transcriptional program of downstream genes such as *RTEL1* and *PORCN*.

Our research identified the distinct remodeling patterns of immune homeostasis following haplo‐SCT and MSDT. Allografts consist of a mixture containing both donor‐derived mature T cells and hematopoietic progenitors, regardless of whether the transplantation involves peripheral blood or bone marrow cells, either separately or in combination. By utilizing transplantation mouse models and conducting clonotype analysis on clinical T cell subsets, our research uncovered that CD8 T cells were preferentially expanded through the peripheral pathway following HSCT, and led to GVHD damage in haplo‐SCT recipients. In haplo‐SCT recipients who achieved immune homeostasis, their CD8 T cells were successfully reconstituted from the central pathway with the help of clinical interventions. While in MSDT, it was observed that a considerable number of CD8 T cells reconstituted from the peripheral pathway, resulting in limited TCR repertoire diversity and potentially leading to the high relapse rate observed in certain conditions in MSDT.^[^ ^26^ ^]^ Therefore, promoting thymus‐dependent central T cell regeneration pathway should also be taken into consideration in MSDT patients.

ATG is an important conditioning regimen that has been proved to induced tolerance in solid organ transplantation,^[^ ^27^ ^]^ and it has become a standard part of conditioning regimens for haplo‐SCT. To investigate whether ATG is involved in different immune homeostasis remodeling patterns in haplo‐SCT and MSDT, we analyzed the immune subtype reconstitution atlas in MSDT recipients with or without ATG conditioning. CD8 Trp cells were expanded in recipients with ATG conditioning, which is consistent with that in haplo‐SCT recipients. This finding suggested ATG may promote the expansion of CD8 Trp cells and contribute to the remodeling of immune homeostasis after HSCT. CD8 Teff cells were expanded in recipients without ATG conditioning, indicating ATG might deplete CD8 Teff cells after MSDT. However, the clonotype of CD8 Teff cells showed no difference between recipients with and without ATG conditioning, and there still existed a proportion of consistent clonotype CD8 Teff cells in recipients with ATG conditioning. These results suggested that the differential bias in T cell reconstitution pathways is not attributed to ATG conditioning. Instead, it appears to be primarily influenced by the HLA disparity between the donor and recipient. These results provide valuable insight into the dynamic patterns of immune homeostasis remodeling following haplo‐SCT and MSDT, and highlight the intrinsic differences of T cell reconstitution biases between these two transplantation approaches. It prompts further investigation in the underlying mechanisms driving immune homeostasis remodeling after HSCT, and paves the way for personalized therapeutic strategies to optimize immune restoration and enhance patient outcomes in the context of allo‐HSCT.

## 4 Experimental Section

### Clinical Samples

*Sequencing Samples in Discovery Cohort*: Peripheral blood samples were collected from 14 donor‐recipient pairs. scRNA‐seq and scTCR‐seq of purified PBMCs were performed from 9 pairs of MSDT and 4 pairs of haplo‐SCT donor recipients. Additionally, to capture more CD8 T cells, CD3^+^CD8^+^ T cells were sorted from one donor‐recipient pair of haplo‐SCT and performed scRNA‐seq and scTCR‐seq (Table S1, Supporting Information). CD3^+^CD4^+^ T cells and CD3^+^CD8^+^ T cells were sorted and performed RNA‐seq and ATAC‐seq. The clinical characteristics of recipients and their paired donors are listed in Table S2 (Supporting Information).

*Spectrum Flow Cytometry and qPCR Samples in Validation Cohort*: Peripheral blood samples were collected from 20 healthy individuals, 12 haplo‐SCT recipients, and 9 MSDT recipients. The spectrum flow cytometry of PBMCs and qPCR of purified CD8 T cells was performed. The clinical characteristics of recipients and their allo‐HSCT related information are listed in Table S6 (Supporting Information).

This study was approved by the Ethics Committee of Peking University People's Hospital with approval number 2020PHB067, and written informed consent was obtained from all subjects in accordance with the Declaration of Helsinki.

### Mice

C57BL/6 (CD45.2, H‐2^b^) and CB6F1 (CD45.2, H‐2^b/d^) mice (8‐10 weeks old) were purchased from Beijing Vital Laboratory Animal Technology Company, Ltd. (Beijing, China). C57BL/6 (CD45.1, H‐2^b^) mice were purchased from Peking University Health Science Center Department of Laboratory Animal Science (Beijing, China). Rosa26‐CAG‐EGFP (C57BL/6J background) mice were purchased from Shanghai Model Organisms Center (Shanghai, China). All mice were maintained in the specific pathogen‐free animal facility of Peking University People's Hospital. This study was approved by the Ethics Committee of Peking University People's Hospital with approval number 2020PHE006. All experiments were performed according to the National Institutes of Health's Guide for the Care and Use of Laboratory Animals.

### Single‐Cell Sequencing by 10× Genomics (RNA and VDJ Sequencing)

PBMCs were isolated by Ficoll density centrifugation. Then, the cells were washed twice with Ca^2+^‐ and Mg^2+^‐free PBS (Gibco, C14190500BT). Single‐cell RNA and VDJ sequencing (scRNA&VDJ‐seq) were performed by the 10× Genomics Chromium platform. Specifically, four scRNA libraries of PBMC samples from 2 donor‐recipient pairs in the haplo‐SCT group were constructed using the Chromium Next GEM Single Cell 3′ Kit v3.1 (1 000 268) and the Chromium Next GEM Chip G Single Cell Kit (1 000 120). The other scRNA&VDJ libraries were prepared with the Chromium Next GEM Single Cell 5′ Kit v2 (1 000 263), the Chromium Next GEM Chip K Single Cell Kit (1 000 286), Library Construction Kit (1 000 190) and the Chromium Single Cell Human TCR Amplification Kit (1 000 252) according to the manufacturer's instructions. Sequencing was performed on an Illumina NovaSeq platform with a paired‐end 150 bp (performed by Berry Genomics Corporation, Beijing, China).

### Single‐Cell RNA‐Seq Data Preprocessing, Integration, and Cell Cluster Identification

Raw sequencing datasets were processed according to the standard Chromium's Cell Ranger pipeline (version 7.1.0) using the human reference version GRCh38 (as provided by 10×) for alignment. The gene expression matrices of cells were filtered by Cell Ranger and analyzed with the workflow in scanpy (version 1.9.1).^[^ ^28^ ^]^ First, quality control was performed to filter low‐quality cells and low‐expressed genes. Cells with 500–4000 detected genes, <20 000 counts, and <15% mitochondria‐associated genes were retained for further analyses. And Genes expressed in at least 3 cells were kept. Scrublet^[^ ^29^ ^]^ were used to predict doublets in each sample separately with default parameters. Next, to normalize the raw count in each cell, a library‐size correction method was applied by using *normalize_total* function (UMI total count per cell was set to 10^4^), and the normalized raw count matrix was saved in the layers named *counts*. Then the count matrix with log1p transformed normalized was deposited in the layers named *logcounts*.

Subsequently, dataset integration and batch effect (sample ID) removal analysis was performed using scvi‐tools (version 0.19.0).^[^ ^30^ ^]^ The top 2000 highly variable genes (HVGs) across batches were identified using scanpy.*pp.highly_variable_genes* function. And the raw counts of these genes were used as the input to the varational autoencoder based integration model (scVI model). The scVI model was trained for 33 unsupervised epochs with default parameters. The resulting 10D latent embeddings (deposited as *X_scVI*) were applied to construct a k‐nearest neighbor graph (KNN). For visualization, cells were projected in two dimensions with the UMAP algorithm with the KNN graph.

Leiden was used, an unsupervised graph‐based clustering algorithm, with resolution 2.1 for cluster identification, which named as *Leiden_2.1*. To cluster cells by the expression matrix, the expression distribution of classic marker genes (e.g., *CD3D*, *CD4*, *CD8A*, *TRDV2*, *CCR7*, *FOXP3*, *CD69*, *FGFBP2*, *GZMK*, *MS4A1*, *CD14*, *CD34*, *PPBP* etc.) was first observed with the *scanpy.pl.umap* function. And the cluster‐specific marker genes of *Leiden_2.1* were calculated using logistic regression with scanpy.*tl.rank_genes_groups* function. In conjunction with the aforementioned steps, the cells that exhibited co‐expression of PPBP and other linage‐specific genes, indicative of potential doublets, were further removed from the analysis. Clusters with similar expression pattern were merged manually. Clusters of differential expression of classic marker genes were extracted and further separated via Leiden algorithm in the second round. Finally, 30 distinct cell types were defined in the dataset.

In addition, cells that were not assigned to CD8‐positive T‐cell clusters were removed from the paired donor‐recipient of CD8 T cell sorted samples. Meanwhile, the processed dataset from Scanpy was converted to R objects using the *LoadH5Seurat* function from SeuratDisk for additional analysis and plots.

### Trajectory Inference

Single‐cell trajectory and pseudotime analysis were conducted using partition‐based graph abstraction (PAGA)^[^ ^31^ ^]^ with *scanpy.tl.paga* function. Specifically, a diffusion map^[^ ^32^ ^]^ with 15 components were calculated to denoise the data by using *scanpy.tl.diffmap* function. And then, another KNN graph (k = 15 neighbors) was constructed with the resulting components from diffusion map. Cluster connectivity was finally calculated using PAGA with default parameters. PAGA plots were constructed with threshold = 0.03. The diffusion pseudotime method (dpt)^[^ ^33^ ^]^ was applied to calculate the pseudotime on the basis of PAGA connectivity predictions.

### Differentially Expressed Genes (DEGs) Identification and GO Enrichment

With the logarithmic normalized count matrix, marker genes for each cell cluster or the DEGs of the same cell type across different sample groups were identified using wilcoxon test. Genes with a fold change (log2) > 0.1 and an adjusted *p* value < 0.05 were used for GO enrichment analysis using the R package topGO. The counts of DEGs in each group for GO enrichment analysis calculated according to the above criterions was no more than 300 in this data.

### Cell State Score Calculation

The cell cycle phase score was calculated with the *CellCycleScoring* function in the R package Seurat^[^ ^34^ ^]^ using the cell cycle markers from Tirosh et al.^[^ ^35^ ^]^ To elucidate the biological process of each cell, ssGSEA analyses were performed on cells with C5: GOBP gene sets downloaded from the Molecular Signatures Database v7.4 (MsigDB) using the R package escape (https://github.com/ncborcherding/escape). To determine the characteristics of each cell subtype, the top 10 marker genes for each CD4 or CD8 T‐cell subtype across all CD4 or CD8 T‐cell types (except for the T‐cell receptor‐associated genes) were integrated. A program score (UCell score) for each cell was assigned using the R package UCell^[^ ^36^ ^]^ with the top 10 genes described above.

### Definition of X‐Positive Cells by scRNA‐Seq

With reference to the method defined by Takuya et al,^[^ ^37^ ^]^ when more than one read of the X gene was detected in a cell, the cell was regarded as an X‐positive cell. For example, the ZNF683‐positive cells were defined by scRNA‐seq as cells with multiple ZNF683 reads. In addition, a cell was considered to be MHCII‐positive if more than one read was detected in one of the MHCII‐associated genes: CD74, HLA‐DQ, HLA‐DP, and HLA‐DR.

### Single‐Cell TCR V(D)J Data Analysis

The raw reads of scTCR‐seq were processed using the Cell Ranger (v.7.1.0) VDJ pipeline with GRCh38 as a reference. The output files “filtered_contig_annotations.csv” and “clonotypes.csv” with TCR clonotype information were generated by the pipeline and used for the following analysis. For TCR analysis, cells with at least one productive TCR α‐chain (TRA) or β‐chain (TRB) were kept for further analysis. For the recipient and the paired donor, clonotypes were defined with the same complementarity determining region amino acid (CDR3aa) sequence as consistent clonotypes and otherwise unique clonotypes. To estimate the diversity of the TCR repertoire sequenced by scTCR‐seq, the Shannon diversity index was calculated using the R package vegan (v.2.5‐7).

### High‐Dimensional Flow Cytometry Data Analysis

Flow Cytometry Standard (FCS) 3.0 files were imported into FlowJo software (version 10) for analysis. Standard gating strategies were employed to remove aggregated and dead cells, then gated CD3^+^ T cell. All CD3^+^ T cells per sample were subsequently imported in Flowjo, biexponentially transformed, and exported for further analysis in R (version 4.3.0). A total of 2088594 cells from healthy individuals (n = 20), haplo‐SCT recipients (n = 11), and MSDT recipients (n = 9) were labeled with a unique computational barcode for further identification and concatenated in a single matrix by using the R package cytofkit2 (v0.99.8) with the parameter transformMethod = “autoLgcl”. A total of 49234 cells exhibiting abnormal fluorescence intensity values were removed by boxplot.stats with the parameter coef = 2.5. Then 2000 cells were random selected from each sample for further analysis, and limma package (v 3.56.2) was used to remove batch effects. The dataset was analyzed using the “FlowSOM” algorithmic from the cytofkit2 package with the parameter k = 30, and visualized by UMAP.

### Public Datasets for Validation

To compare the immunophenotyping states between tolerant and non‐tolerant patients underwent allo‐HSCT, previous published bulk RNA sequencing datasets of PBMCs were retrieved from Gene Expression Omnibus (GEO) database with accession number GSE150735. The GSM4557689 sample and its paired GSM4557688 sample were removed because three different gene count profiles were provided in the GSM4557689 sample, and the final validation dataset enrolled 36 primary tolerant recipients, 20 secondary tolerant recipients, and 37 non‐tolerant recipients and their paired donors, respectively.

### Deconvolution of the Bulk RNA‐Seq Data by AutoGeneS

The public bulk RNA‐seq data of PBMCs were deconvolved into the 30 cell types identified in the study according to the PBMCs scRNA‐seq reference profiles using AutoGeneS (v1.0.4)^[^ ^38^ ^]^ and non‐negative least squares (NNLS). First, the logarithmized normalized count matrix was inputted to measure the centroids of the 30 cell types by means of averaging. Next, during the optimization process, AutoGeneS was employed to generate 5000 solutions with a set of 400 marker genes selected out of the 2000 HVGs (parameters: ngen = 5000, nfeatures = 400, seed = 0, mode = “fixed”). And the solution which minimizes the correlation between cell types was chosen (parameters: index = 1). Finally, bulk RNA‐seq samples were deconvolved using NNLS regression as implemented within AutoGeneS.

### Sorting CD3^+^CD4^+^ and CD3^+^CD8^+^ T Cells for Bulk Sequencing

CD3^+^ T cells were purified from PBMCs by CD3 MicroBeads (Miltenyi Biotec, 130‐097‐043) according to the manufacturer's instructions. Cells were stained with surface antibodies against CD4‐APC (BD Pharmingen, 551 980) and CD8‐PE (BD Pharmingen, 557 086). Cells were incubated on ice for 20 min, washed with 3 ml of PBS, and then resuspended in 500 µl of PBS for sorting on a FACS Aria III (BD Biosciences).

### Bulk RNA Sequencing and Data Processing

RNA was extracted using the RNeasy Micro Kit (QIAGEN, 74 004) and purified by NEBNext Oligo d(T)_25_ beads (NEB, E7490). An NEBNext Ultra II RNA Library Prep Kit (NEB, E7770S) was used to generate cDNA libraries. AMPure XP beads (Beckman Coulter, A63881) were used to purify cDNA libraries between 300 and 500 bp. Paired‐end sequencing was performed on a NovaSeq6000 (Illumina) and produced between 28 and 35 million 150‐bp paired‐end reads per sample.

Raw RNA‐seq FASTQ files were processed according to a previously described method.^[^ ^39^ ^]^ Low‐quality reads and adapter sequences were cleaned up, and the remaining reads were quantified against the GRCh38 reference at the transcript level using Salmon software and aggregated at the gene level using the R package tximport (v.1.22.0). The count values were used for DEG calculation with the R packages DESeq2 (v.1.34.0) and IHW (v.1.22.0). DEGs with weighted P values < 0.05 and a fold change (log2) > 0 were used for GO enrichment analysis. For the bulk RNA‐seq of ZNF683‐overexpressing CD8 T cells and control CD8 T‐cell samples, genes with a |correlation coefficient| > 0.4 and a *p* value < 0.05 were regarded as being positively or negatively correlated with ZNF683 and used for subsequent GO enrichment analysis.

### Bulk‐ATAC Sequencing and Data Processing

A total of 5×10^4^ sorted cells were resuspended and lysed in 50 µl of lysis buffer (10 mM NaCl, 10 mM Tris‐HCl, pH 7.4, 3 mM MgCl_2_, 0.1% ICEPALEA‐630) after being washed twice in PBS. DNA was fragmented using a TruePrep DNA Library Prep Kit V2 for Illumina (Vazyme, TD501‐01) and purified by using a MinElute PCR Purification Kit (QIAGEN, 28 006). The purified DNA was barcoded with TruePrep Index Kit V2 for Illumina (Vazyme, TD202) and amplified by PCR using a TruePrep DNA Library Prep Kit V2 for Illumina. VAHTS DNA Clean Beads (Vazyme, N411) were used to purify cDNA libraries between 100 and 1000 bp in size. Paired‐end sequencing was performed on a NovaSeq6000 (Illumina).

Raw ATAC‐seq FASTQ files from paired‐end sequencing were processed according to a previously described method.^[^ ^40^ ^]^ Clean FASTQ files were aligned to the GRCh38 reference genome using Bowtie2. Samtools was used to remove unmapped, unpaired, and mitochondrial reads. PCR duplicates were removed using Picard. Reads were shifted +4 bp and ‐5 bp for positive and negative strands, respectively. Peak calling was performed using MACS2 with FDR q‐value 0.01. The peaks of all samples were combined to create a union peak list and merged overlapping peaks with the BedTools merge command. The number of reads in each peak was determined using BedTools coverage. DA regions were identified following DESeq2 normalization using an FDR cutoff < 0.05. Motif enrichment was calculated using HOMER (default parameters) on peaks that were DA across compared group. Transcription binding site prediction analysis was performed using a known motif discovery strategy.

### Transplantation Mouse Models

Splenic T cells were isolated by negative selection using a Pan T Cell Isolation Kit II (Miltenyi‐Biotec, Germany), and the obtained cells had a purity of >95%. T cell‐depleted bone marrow cells (TCD‐BM) were isolated with anti‐CD90.2 MicroBeads (Miltenyi‐Biotec). Recipient mice received 8 Gy total body irradiation. Combined 5×10^6^ splenic T cells and 5×10^6^ TCD‐BM cells were transplanted intravenously to recipient mice on day 0. The survival of recipient mice was monitored daily, and the body weight was recorded every 5 days.

### Overexpression of ZNF683 in Human Primary T Cells

CD3^+^ T cells were purified from PBMCs by CD3 MicroBeads (Miltenyi Biotec, 130‐097‐043) according to the manufacturer's instructions. T cells were prestimulated with Dynabeads Human T‐Activator CD3/CD28 beads (Invitrogen, 11131D) and 100 U ml^−1^ rhIL‐2 in IMDM medium (Gibco, Invitrogen) containing 10% BIT 9500 (Stemcell Technologies, 0 9500) for 24 h. ZNF683‐overexpressing lentivirus was purchased from Sangon Biotech (Shanghai, China). After 24 h of culture, the cells were transduced with lentiviruses, 6 µg mL^−1^ polybrene (Sigma, USA) was added. The cells were incubated for another 24 h at 37 °C and 5% CO_2,_ and the medium was replaced with fresh medium. GFP^+^ cells were isolated after 72 h of infection and routinely cultured in IMDM containing 10% BIT 9500 with rhIL‐2.

### Flow Cytometry

Surface staining was performed at room temperature (RT) with antibodies for 20 min. Cells were stained with surface antibodies against human CD4 and CD8 (BD Pharmingen) for 20 min at RT. For cytokine detection, T cells were stimulated with phorbol 12‐myristate 13‐acetate (PMA, 50 ng mL^−1^, Sigma Aldrich, 880134P) and ionomycin (1000 ng mL^−1^, Sigma Aldrich, I3909) for 4 h at 37 °C in the presence of GolgiPlug (BD Pharmingen, 555 029). Intracellular staining of IFN‐γ and Ki67 was carried out by using the Transcription Factor Buffer Set kit (BD Pharmingen, 562 574) after being resuspended according to the manufacturer's instructions and then incubated for 20 min with antibodies at RT. Detailed antibody information is listed in Table S7 (Supporting Information).

### T‐Cell Proliferation Analysis

Sorted GFP^+^ T cells were stained with Cell Trace Violet Fluorescent (ThermoFisher, C34557) at a final concentration of 5 µM for 5 min at 37 °C and washed twice with complete medium containing 10% FBS. Labeled T cells (2×10^5^ cells per well) were stimulated with CD3/28 beads in flat‐bottomed 96‐well plates in IMDM containing 10% BIT 9500 with 100 U mL^−1^ rhIL‐2. After 96 h of culture, the cells were harvested, stained with CD4‐PercpCy5.5 and CD8‐APC‐R700, and then analyzed by flow cytometry. Detailed antibody information is listed in Table S7 (Supporting Information).

### Quantitative Real‐Time PCR

RNA was extracted with a RNeasy mini kit (QIAGEN, 74 106) according to the manufacturer's protocol. The amount of RNA was quantified with Qubit (ThermoFisher). Synthesis of cDNA was carried out with a cDNA reverse transcription kit (TaKaRa, RR047A). qPCR was performed with SYBR Green (Roche, 04913914001). A 7500 real‐time PCR system (Applied Biosystems) was used to detect the signal. Values were normalized according to the expression of the housekeeping gene *18S* in the same samples by using the 2^−ΔΔCT^ method. The primer sequences were as follows: ZNF683 forward: 5′‐CATATGTGGCAAGAGCTTTGG‐3′; ZNF683 reverse: 5′‐GGCAAGTTGAGTGAAGCTCT‐3′; PORCN forward: 5′‐GGAGGCTCGGGTTGC‐3′; PORCN reverse: 5′‐GCCCCTCGCATCTTGTGCCA‐3′; RTEL1 forward: 5′‐CCTGAATGGTGTGACCGTAGA‐3′; RTEL1 reverse: 5′‐CCTGTACCCGTAGGGCTCTC‐3′; human 18S forward: 5′‐ACCGATTGGATGGTTTAGTGAG‐3′; and human 18S reverse: 5′‐CCTACGGAAACCTTGTTACGAC‐3′.

### Statistical Analyses

All the results are shown as mean ± SEM. Student's *t* test was used for two groups analyses. One‐way analysis of variance (ANOVA) is used to compare the means of more than two groups. P values<0.05 were considered to be significant. The absence of P values in the graphs indicates that there is no statistic difference between groups. Statistical analyses were performed on GraphPad 8.0 software.

## Conflict of Interest

The authors declare no conflict of interest.

## Author Contributions

H.D.G. collected all clinical samples and performed experiments with the help of B.X.W., X.Y.J., and Z.G.W., L.P.G. wrote the code for data analysis, X.D.M., Y.Q.S., Y.Y.Z., Z.D.W., J.K., and C.H.Y. recruited patients, H.D.G. and L.P.G. wrote the manuscript and prepared the figures under supervision of X.J.H. All authors read and approved the final draft and submission and take full responsibility of its content.

## Supporting information

Supporting Information

Supplemental Table 3

Supplemental Table 4

Supplemental Table 5

## Data Availability

The data that support the findings of this study are openly available in The raw sequencing data generated by this project were deposited into the Genome Sequence Archive (https://ngdc.cncb.ac.cn/gsa‐human) with accession number GSA‐Human: HRA007935. at https://ngdc.cncb.ac.cn/gsa‐human, reference number HRA007935.
