# Supplementary material for: Distinct Immune Homeostasis Remodeling Patterns after HLA‐Matched and Haploidentical Transplantation
Source: Adv Sci (Weinh). 2024 Sep 3;11(39):2400544. doi: 10.1002/advs.202400544 (PMC11497014; doi:10.1002/advs.202400544)
Supplement: Supplementary file 1 — Supporting Information [file ADVS-11-2400544-s004.docx]

**Supporting Information**

Title: Distinct Immune Homeostasis Remodeling Patterns after HLA-matched and Haploidentical Transplantation

Huidong Guo^†^, Liping Guo^†^, Bixia Wang, Xinya Jiang, Zhigui Wu, Xiao-Dong Mo, Yu-Qian Sun, Yuan-Yuan Zhang, Zhi-Dong Wang, Jun Kong, Chen-Hua Yan, and Xiao-Jun Huang^*^


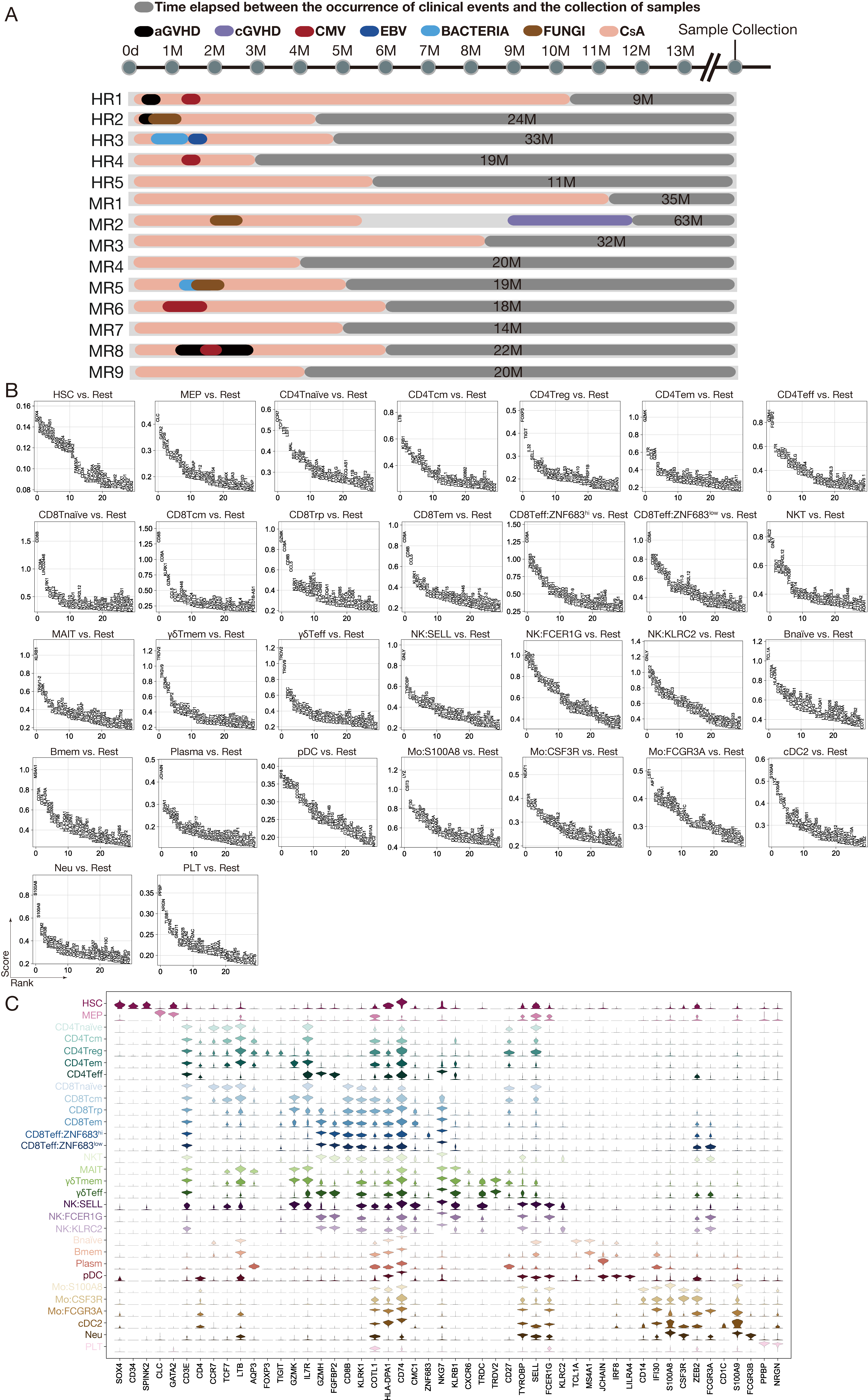


**Figure S1. Clinical information of samples in sequencing analysis from discovery cohort and gene expression levels in identified clusters.**

**(A)** The occurrence time of clinical events including acute GVHD (aGVHD), chronic GVHD (cGVHD), Cytomegalovirus (CMV) infection, Epstein-Barr virus (EBV) infection, bacteria and fungi infection in patients after HSCT. Systemic immunosuppressants usage timeline of cyclosporin A (CsA) in patients after allo-HSCT. The interval between the end of clinical events and sample collection time in recipients after allo-HSCT. HR, haplo-SCT recipient; MR, MSDT recipient. **(B)** The top 30 genes of each identified cell subpopulation were ranked by their predictive weights (scores) as estimated by logistic regression. **(C)** The violin plot shows the expression patterns of the signature genes in each cell subpopulations.


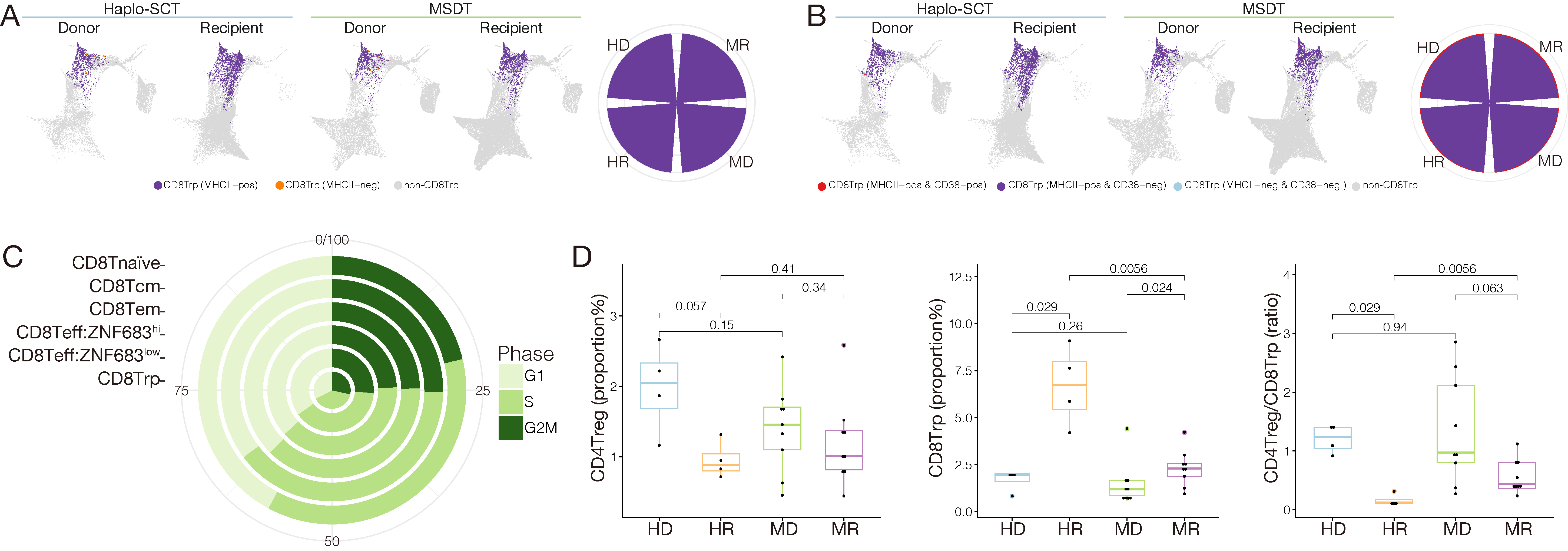


**Figure S2. Transcriptome program of CD8 Trp cells.**

**(A)** The distribution of MHCII-positive CD8 Trp cells (more than one read of MHCII-associated genes) in the indicated groups visualized with PAGA plot. Purple represents MHCII-positive CD8 Trp cells, and orange represents MHCII-negative CD8 Trp cells. **(B)** The distribution of MHCII-associated genes and CD38 in CD8 Trp cells. Red represents both MHCII- and CD38-positive (more than one read) CD8 Trp cells, purple represents MHCII-positive and CD38-negative CD8 Trp cells, and blue represents double-negative CD8 Trp cells. **(C)** Donut plots shows the percentages of cells in G1, S, and G2M phases in CD8 T-cell subpopulations. **(D)** Quantiﬁcation of CD4 Treg and CD8 Trp cell frequency among recipients and paired donors from the haplo-SCT or MSDT group. HD, haplo-SCT donor; HR, haplo-SCT recipient; MD, MSDT donor; MR, MSDT recipient.


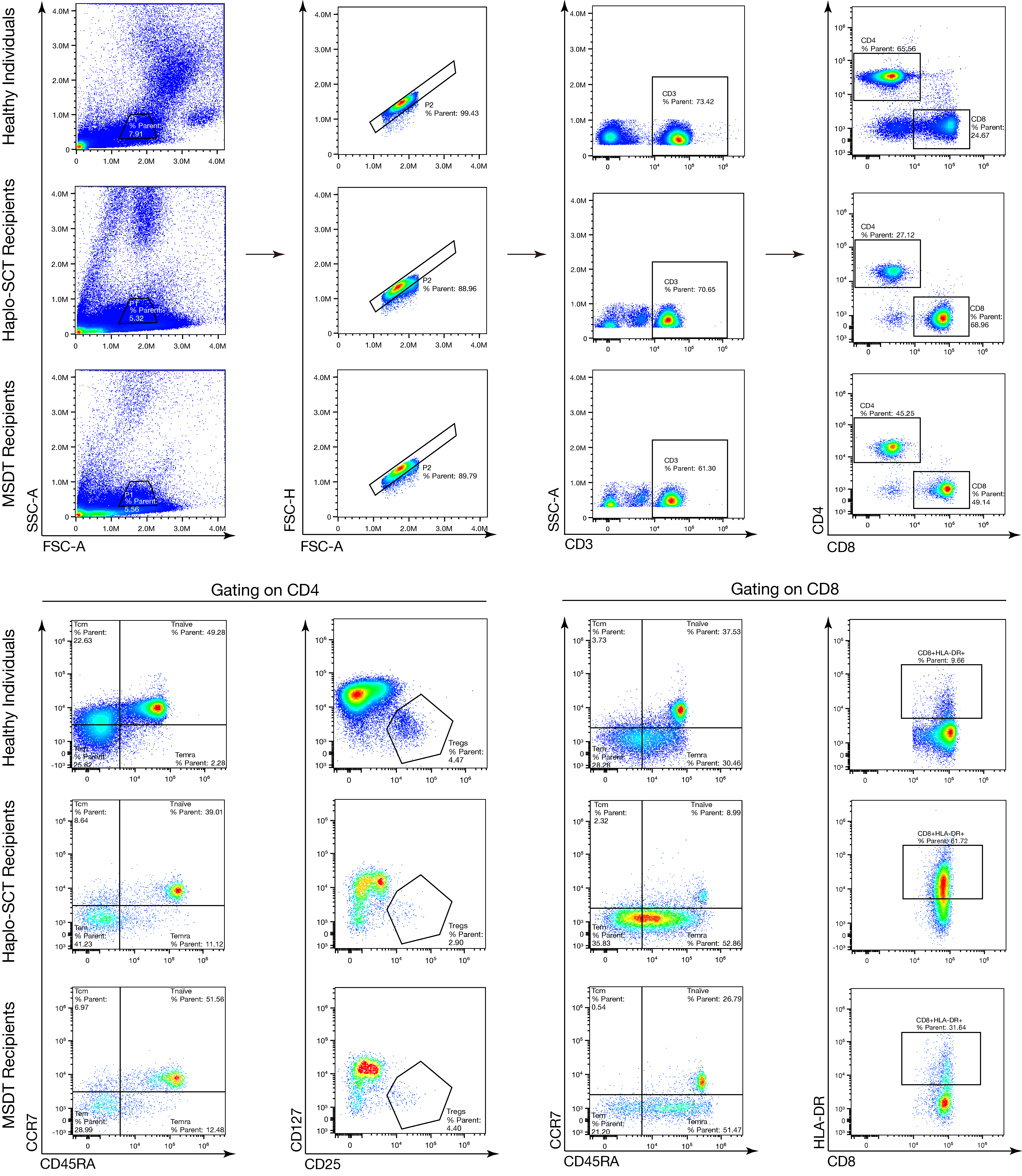


**Figure S3. Gating strategy for spectrum flow cytometry detecting T cell reconstitution in recipients after allo-HSCT.**

**
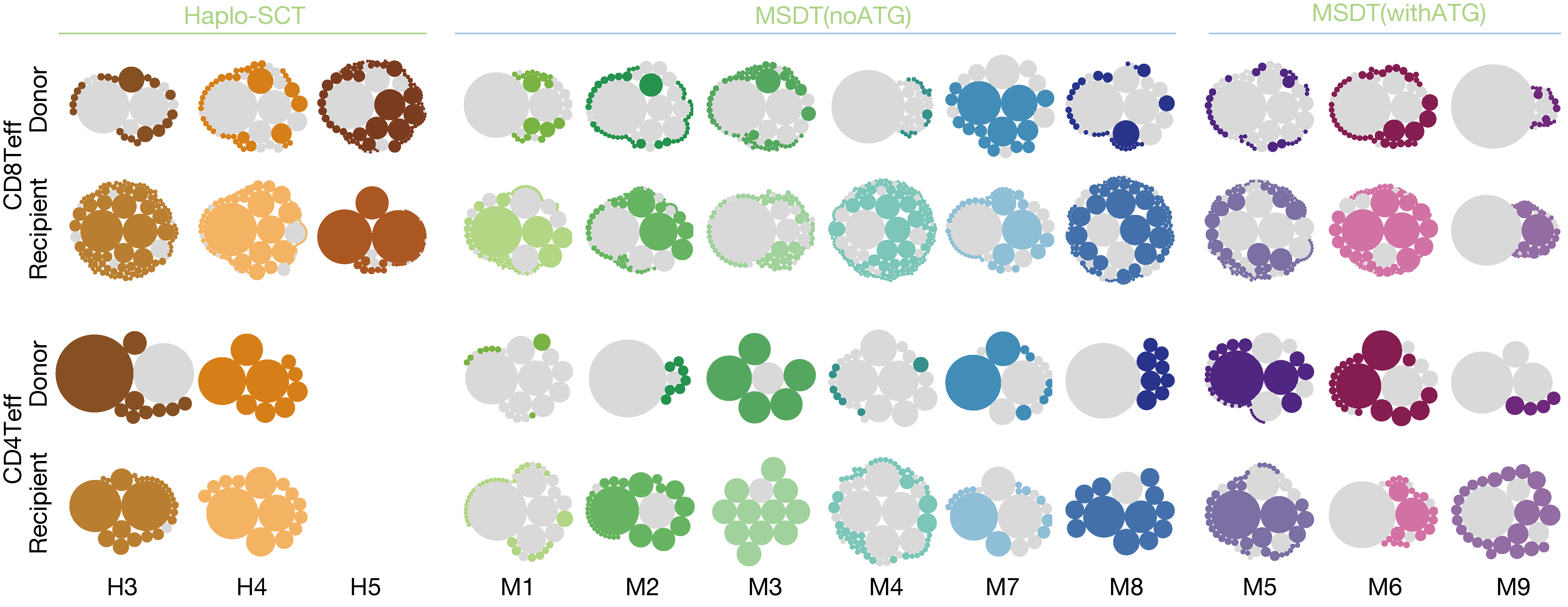
**

**Figure S4. Distribution of CD4 Teff and CD8 Teff clonotypes in paired donor-recipient from haplo-SCT and MSDT groups.**

**
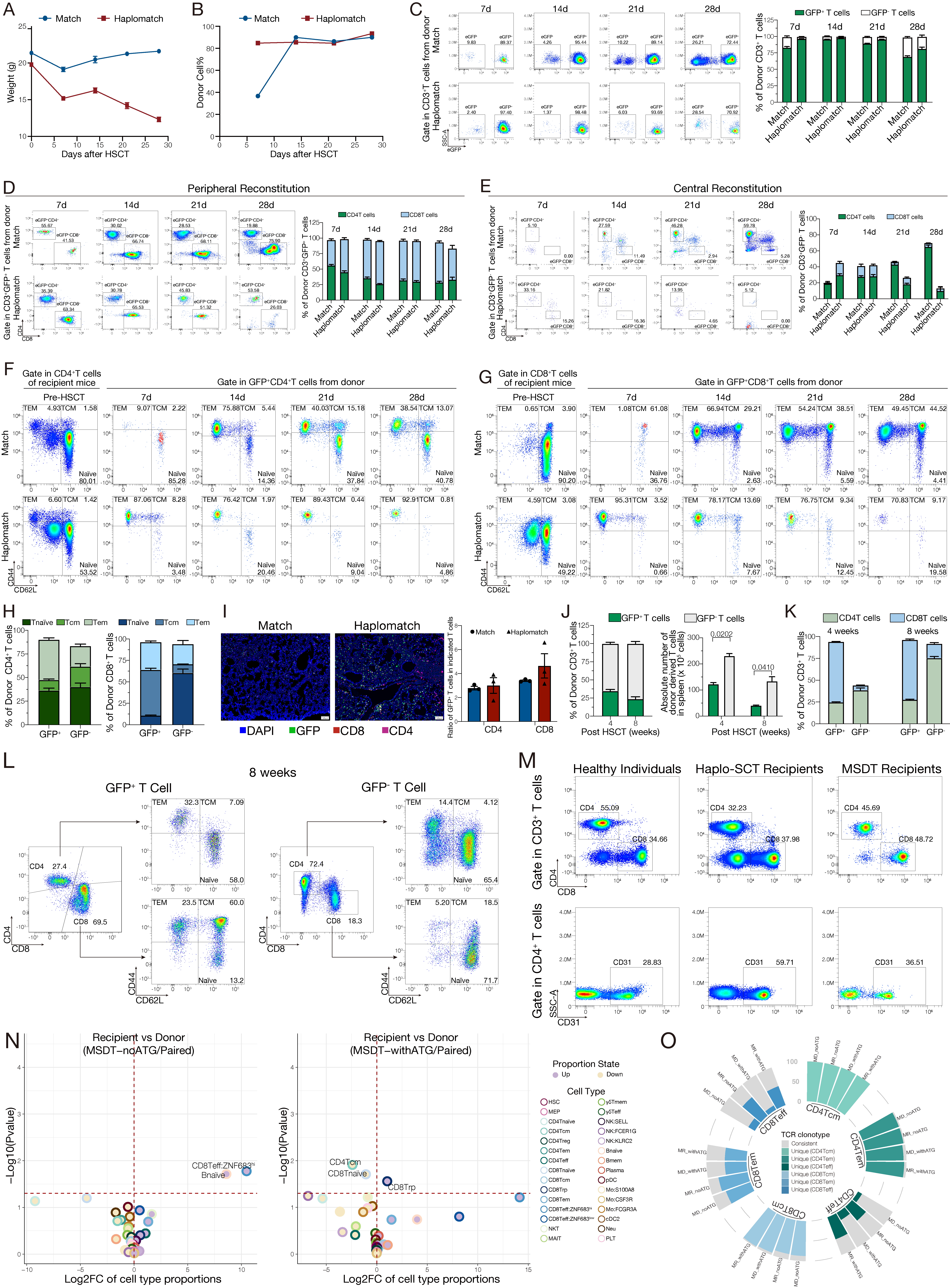
**

**Figure S5. Dynamics of T cell reconstitution from the peripheral and central pathways in MHC matched and haplomatched transplantation models.**

**(A)** Weight of MHC-match and MHC-haplomatch recipient mice after transplantation. **(B)** Chimerism rate of donor cells in MHC-match and MHC-haplomatch recipient mice after transplantation. **(C)** The representative flow cytometry graph and ratio of T cells that reconstitute from the peripheral (GFP^+^) and central (GFP^-^) pathway in peripheral blood of MHC-match and MHC-haplomatch recipient mice after transplantation. **(D-E)** The representative flow cytometry graph and ratio of CD4 and CD8 T cells that reconstitute from the peripheral (D, GFP^+^) and central (E, GFP^-^) pathway in peripheral blood of MHC-match and MHC-haplomatch recipient mice after transplantation. **(F-G)** Representative flow cytometry graph of T cell subsets that reconstitute from the peripheral pathway in peripheral blood of MHC-match and MHC-haplomatch recipient mice after transplantation. **(H)** Ratio of donor-derived T cell subsets that reconstitute from the peripheral (GFP^+^) and central (GFP^-^) pathway in spleen of MHC-match and MHC-haplomatch recipient mice at 4 weeks after transplantation. **(I)** T cell infiltration in lung of MHC-match and MHC-haplomatch recipient mice at 4 weeks after transplantation. 3 mice per group. **(J)** The ratio and absolute number of donor-derived T cells that reconstitute from the peripheral (GFP^+^) and central (GFP^-^) pathway in spleen of MHC-match recipient mice at 4 weeks and 8 weeks after transplantation. **(K)** The ratio of CD4 and CD8 T cells that reconstitute from the peripheral (GFP^+^) and central (GFP^-^) pathway in spleen of MHC-match recipient mice at 4 weeks and 8 weeks after transplantation. **(L)** Representative flow cytometry graph of T cells that reconstitute from the peripheral (GFP^+^) and central (GFP^-^) pathway in spleen of MHC-match recipient mice at 8 weeks after transplantation. **(M)** Representative flow cytometry graph of the expression level of CD31 in CD4 T cells from healthy individuals, haplo-SCT recipients and MSDT recipients. **(N)** Quantiﬁcation of cell cluster frequency among MSDT recipients with ATG conditioning and without ATG conditioning, compared to their paired donors respectively. The increased and decreased cell types are shown in purple and light brown, respectively. The horizontal red dashes indicate the boundary of a P value equal to 0.05. Paired limma test were used for comparisons between donors and recipients. **(O)** Clonotype of indicated T cells in paired donor-recipient from MSDT group with or without ATG conditioning. MD, MSDT donor; MR, MSDT recipient. Unpaired *t*-test. At least 5 mice per group in the assessment of immune reconstitution in peripheral blood. 3 mice in MHC-match group in the assessment of immune reconstitution in spleen. Combined data from two independent experiments. The symbols represent individual mice. Error bars represent the mean ± SEM.

**
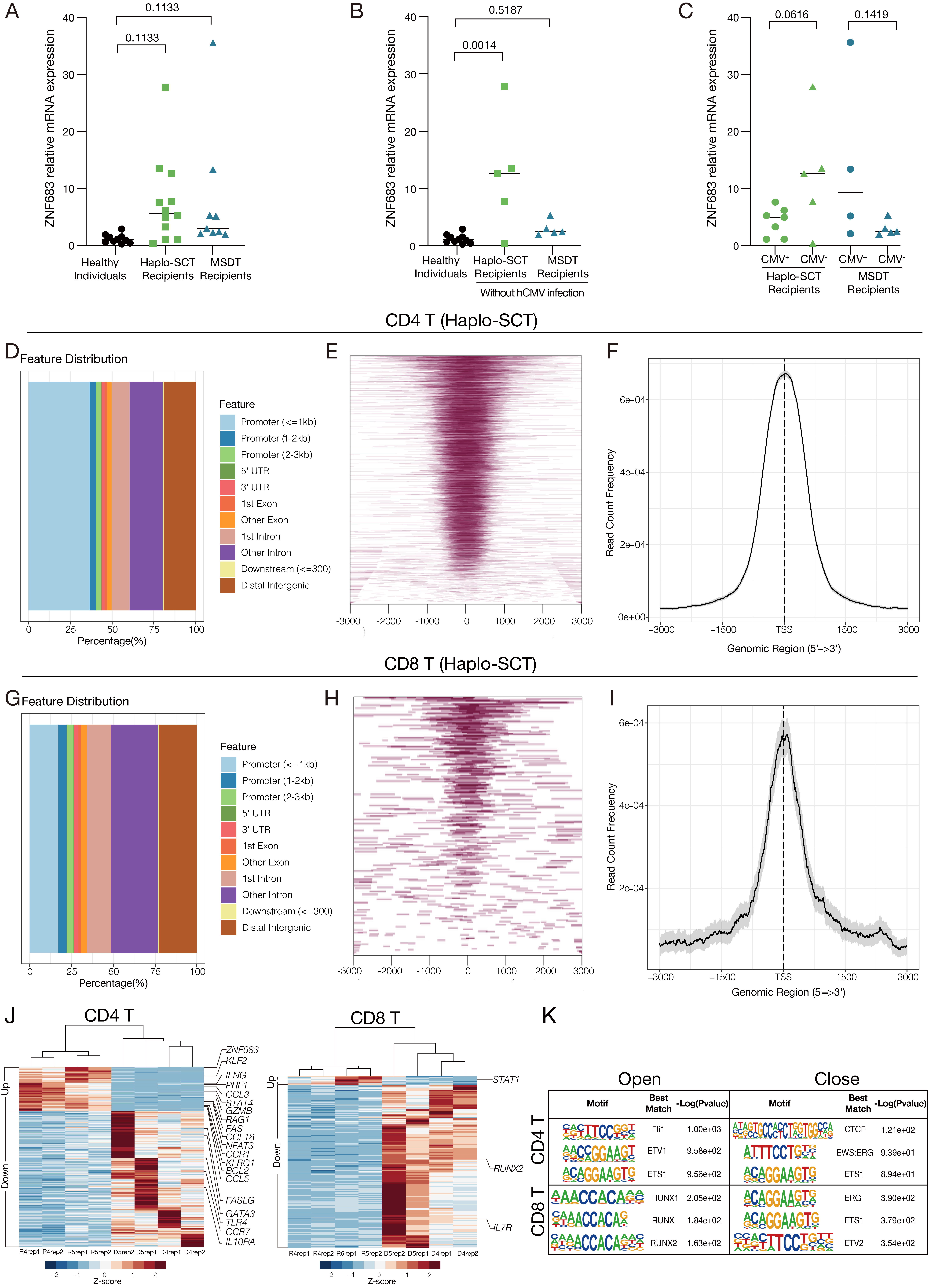
**

**Figure S6. *ZNF683* expression level in T cells and T cell chromatin accessibility from haplo-SCT recipients.**

**(A)** *ZNF683* expression level in CD8 T cells from healthy individuals (N=10), haplo-SCT recipients (N=12) and MSDT recipients (N=9). One-way-ANOVA. **(B)** *ZNF683* expression level in CD8 T cells from healthy individuals (N=10), haplo-SCT recipients without hCMV infection (N=5) and MSDT recipients without hCMV infection (N=5). One-way-ANOVA. **(C)** *ZNF683* expression level in CD8 T cells from haplo-SCT recipients or MSDT recipients that with or without hCMV infection. Unpaired *t*-test. **(D)** DA peak annotation in the entire genome for CD4^+^ T cells in haplo-SCT recipients compared with their paired donors. **(E)** DA peak distributions around transcription start sites (TSSs) for CD4^+^ T cells in haplo-SCT recipients compared with their paired donors. **(F)** Aggregate plot showing DA peaks around TSSs for CD4^+^ T cells in haplo-SCT recipients compared with their paired donors. **(G-I)** The same analyses as shown in (D)-(F), respectively, but for the CD8^+^ T cells. **(J)** Heatmap shows different accessibility-proximal genes in CD4 and CD8 T cells from haplo-SCT recipients compared with their paired donors. FDR q-value < 0.05. **(K)** Top 3 enriched TF motifs in the DA peaks of CD4 and CD8 T cells are shown.

**
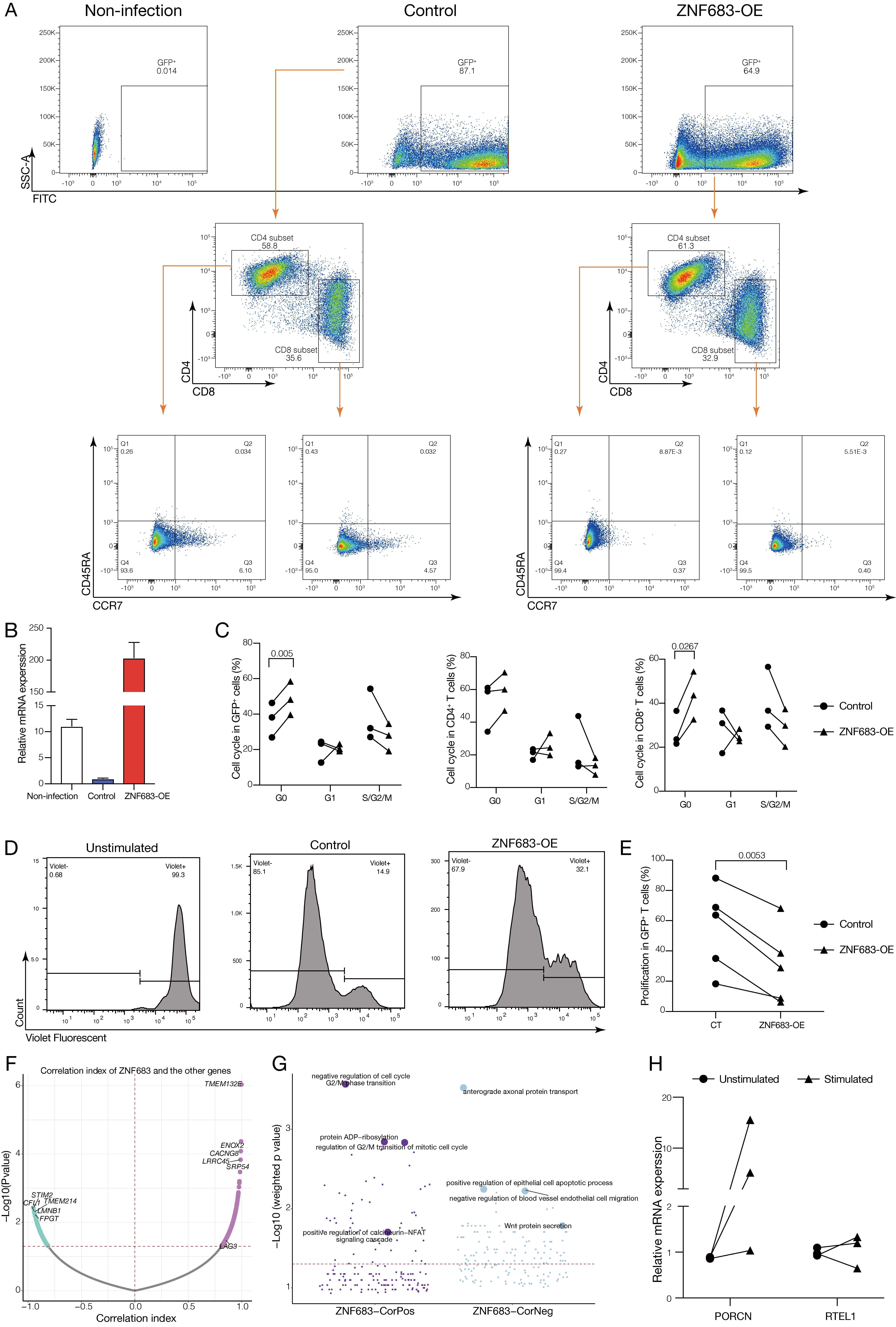
**

**Figure S7. Overexpression of *ZNF683* in human primary T cells.**

**(A)** Gating strategy for ZNF683-overexpressing human primary T cells. **(B)** qPCR was used to validate the expression level of ZNF683. **(C)** Comparison of the percentages of G0, G1 and S/G2/M in GFP^+^ cells, GFP^+^CD4^+^ cells and GFP^+^CD8^+^ cells in the control and ZNF683-overexpressing groups. **(D-E)** Cell Trace Violet analysis showed the proliferation of T cells in the unstimulated, control and ZNF683-overexpressing groups. **(F)** Genes that correlated with ZNF683 in CD8 T cells. **(G)** Enriched GO terms in ZNF683-positive correlated genes and ZNF683-negative correlated genes. **(H)** *PORCN* and *RTEL1* expression level in CD8^+^ T cells from haplo-SCT recipients who have rebalanced immune homeostasis, stimulated or unstimulated with CD3/CD28 microbeads. Two-tailed paired t-test.

**Supplemental Tables**

**Table S1. Sample information for sequencing analysis in discovery cohort.**

| **Sample ID** | **Disease** | **HSCT Type** | **Donor-Recipient HLA mismatches** | **Single Cell Sequencing Cell type** | **10× scRNAseq** | **10× scTCRseq** | **ATAC-seq** | **RNA-**  **seq** |
| --- | --- | --- | --- | --- | --- | --- | --- | --- |
| HD1/HR1 | AML | HLA-Haploidentical | A、B、DRB1(3/6) | PBMCs | **🗸** |  |  | **🗸** |
| HD2/HR2 | AML | HLA-Haploidentical | A (5/6) | PBMCs | **🗸** |  | **🗸** | **🗸** |
| HD3/HR3 | AML | HLA-Haploidentical | A、B、DRB1(3/6) | PBMCs | **🗸** | **🗸** | **🗸** | **🗸** |
| HD4/HR4 | AML | HLA-Haploidentical | 1. B、DRB1(3/6) | PBMCs | **🗸** | **🗸** | **🗸** | **🗸** |
| HD5/HR5 | MDS | HLA-Haploidentical | A、B、DRB1(3/6) | CD8^+^ T cells | **🗸** | **🗸** |  |  |
| MD1/MR1 | MDS | HLA-identical | - | PBMCs | **🗸** | **🗸** | **🗸** | **🗸** |
| MD2/MR2 | MDS | HLA-identical | - | PBMCs | **🗸** | **🗸** | **🗸** | **🗸** |
| MD3/MR3 | AML | HLA-identical | - | PBMCs | **🗸** | **🗸** | **🗸** | **🗸** |
| MD4/MR4 | AML | HLA-identical | - | PBMCs | **🗸** | **🗸** |  |  |
| MD5/MR5 | AML | HLA-identical | - | PBMCs | **🗸** | **🗸** |  |  |
| MD6/MR6 | AML | HLA-identical | - | PBMCs | **🗸** | **🗸** |  |  |
| MD7/MR7 | MDS | HLA-identical | - | PBMCs | **🗸** | **🗸** |  |  |
| MD8/MR8 | MDS | HLA-identical | - | PBMCs | **🗸** | **🗸** |  |  |
| MD9/MR9 | AML | HLA-identical | - | PBMCs | **🗸** | **🗸** |  |  |

**Abbreviations:** HD, haplo-SCT donor; HR, haplo-SCT recipient; MD, MSDT donor; MR, MSDT recipient; Haplo-SCT, haploidentical stem cell transplantation; MSDT, HLA-matched sibling donor transplantation; AML, acute myelogenous leukemia; MDS, myelodysplastic syndromes; PBMCs, peripheral blood mononuclear cells; scRNA-seq, single-cell RNA sequencing; scTCR-seq, single-cell TCR sequencing; RNA-seq, RNA sequencing; ATAC-seq, Assay for Transposase-Accessible Chromatin with high throughput sequencing.

**Table S2. Patient and donor characteristics for sequencing sample in discovery cohort.**

| **Characteristics** | Sequencing | |  |
| --- | --- | --- | --- |
|  | Haplo-SCT  **(N=5)** | MSDT  **(N=9)** | *P*-Value* |
| Interval of sample collection and HSCT (range), months | 23(18-59) | 25(23-75) | 0.360 |
| Patient age at HSCT (range) | 55(38-55) | 46(25-57) | 0.54 |
| Patient gender (male/female) | 3/2 | 4/5 | 1 |
| Underlying disease |  |  | 0.580 |
| AML | 4 | 5 |  |
| MDS | 1 | 4 |  |
| Source of allografts |  |  | 0.031 |
| PB+BM | 5 | 3 |  |
| PB | 0 | 6 |  |
| Donor age | 28(18-63) | 50(22-60) | 0.317 |
| Donor gender (male/female) | 1/4 | 5/4 | 0.310 |
| Donor-recipient gender |  |  |  |
| Female to male | 2 | 2 | 0.496 |
| Female to female | 2 | 2 |  |
| Male to male | 1 | 2 |  |
| Male to female | 0 | 3 |  |
| Donor-recipient blood type |  |  | 1.0 |
| Match | 3 | 6 |  |
| Mismatch | 2 | 3 |  |
| Pre-HSCT cycles of chemotherapy | 4 (2-6） | 3 (0-5) | 0.331 |
| Pre-HSCT conditioning |  |  |  |
| BU/CY+ATG | 5 | 3 | 0.031 |
| BU/CY | 0 | 6 |  |
| HCMV infection after HSCT |  |  | 1.0 |
| Positive | 3 | 4 |  |
| Negative | 2 | 5 |  |
| Graft Composition |  |  |  |
| Transplanted total nucleated cell dose (×10^8^/ kg) | 8.52(6.55-9.71) | 7.89(5.90-11.64) | 0.898 |
| Transplanted BM nucleated cell dose (×10^8^/ kg) | 2.83(1.08-3.08) | 0.00(0.00-3.60) | 0.118 |
| Transplanted PB nucleated cell dose (×10^8^/ kg) | 6.22(3.72-6.79) | 6.46(3.70-11.64) | 0.179 |
| Transplanted CD34^+^ cell dose (×10^6^/ kg) | 2.55(1.28-3.99) | 2.16(1.99-5.16) | 0.298 |
| Transplanted CD3^+^ cell dose (×10^8^/ kg) | 1.78(1.08-3.76) | 1.73(0.84-4.84) | 1.0 |
| Transplanted CD4^+^ cell dose (×10^8^/ kg) | 1.23(0.55-1.60) | 0.87(0.23-1.39) | 0.112 |
| Transplanted CD8^+^ cell dose (×10^8^/ kg) | 0.54(0.18-1.44) | 0.67(0.10-0.89) | 0.791 |
| Transplanted CD14^+^ cell dose (×10^8^/ kg) | 1.24(0.50-1.65) | 1.22(1.17-2.69) | 0.898 |

**Abbreviations:** *Continuous variables were compared using the Mann-Whitney U test; categorical variables were compared using Fisher's exact test. P<0.05 was considered significant. HSCT, hematopoietic stem cell transplantation; Haplo-SCT, haploidentical stem cell transplantation; MSDT, HLA-matched sibling donor transplantation; AML, acute myelogenous leukemia; MDS, myelodysplastic syndromes; PB, peripheral blood; BM, bone marrow; BU/CY, busulfan and cyclophosphamide; ATG, antithymocyte globulin; hCMV, Human cytomegalovirus.

**Table S6. Patient characteristics and their allo-HSCT related information for spectrum flow cytometry and qPCR samples in validation cohort.** (Related to Figure 3F-H, Figure 5N, Figure S3 and Figure S6A-C)

| **Characteristics** | Spectrum flow cytometry and qPCR | | |
| --- | --- | --- | --- |
|  | Haplo-SCT  (N=12) | MSDT  (N=9) | *P*-Value* |
| Interval of sample collection and HSCT (range), months | 15 (12-20) | 16 (10-19) | 0.348 |
| Patient median age at HSCT (range), years | 33 (19-57) | 48 (24-55) | 0.069 |
| Patient gender (male/female) | 4/8 | 4/5 | 0.673 |
| Underlying disease |  |  | 0.611 |
| AML | 10 | 6 |  |
| MDS | 2 | 3 |  |
| Source of allografts |  |  | 0.486 |
| PB+BM | 2 | 0 |  |
| PB | 10 | 9 |  |
| Donor age | 34 (17-59) | 49 (21-57) | 0.109 |
| Donor gender (male/female) | 5/7 | 4/5 | 1.000 |
| Donor-recipient gender |  |  | 0.738 |
| Female to male | 3 | 3 |  |
| Female to female | 2 | 1 |  |
| Male to male | 1 | 2 |  |
| Male to female | 6 | 3 |  |
| Donor-recipient blood type |  |  | 0.660 |
| Match | 6 | 6 |  |
| Mismatch | 6 | 3 |  |
| Pre-HSCT conditioning |  |  | 0.006 |
| BU/CY+ATG | 12 | 4 |  |
| BU/CY | 0 | 5 |  |
| hCMV infection after HSCT |  |  | 0.670 |
| positive | 7 | 4 |  |
| negative | 5 | 5 |  |
| Graft Compositions |  |  |  |
| Transplanted total nucleated cell dose (×10^8^/ kg) | 9.77 (8.12-13.28) | 9.75 (4.82-18.63) | 0.732 |
| Transplanted BM nucleated cell dose (×10^8^/ kg) | 2.56 (2.13-2.99) | 0.00 |  |
| Transplanted PB nucleated cell dose (×10^8^/ kg) | 9.63 (5.99-13.28) | 9.75 (4.82-18.63) | 0.970 |
| Transplanted CD34^+^ cell dose (×10^6^/ kg) | 4.27 (1.10-14.10) | 3.72 (2.05-9.32) | 0.621 |
| Transplanted CD3^+^ cell dose (×10^8^/ kg) | 3.48 (2.52-6.98) | 2.42 (0.86-6.15) | 0.138 |
| Transplanted CD4^+^ cell dose (×10^8^/ kg) | 1.99 (1.09-4.31) | 1.45 (0.49-3.27) | 0.271 |
| Transplanted CD8^+^ cell dose (×10^8^/ kg) | 1.43 (0.60-2.63) | 0.88 (0.32-2.35) | 0.053 |
| Transplanted CD14^+^ cell dose (×10^8^/ kg) | 1.88 (0.96-2.72) | 1.57 (0.99-4.40) | 0.849 |

**Abbreviations:** *Continuous variables were compared using the Mann-Whitney U test; categorical variables were compared using Fisher's exact test. P<0.05 was considered significant. HSCT, hematopoietic stem cell transplantation; Haplo-SCT, haploidentical stem cell transplantation; MSDT, HLA-matched sibling donor transplantation; AML, acute myelogenous leukemia; MDS, myelodysplastic syndromes; PB, peripheral blood; BM, bone marrow; BU/CY, busulfan and cyclophosphamide; ATG, antithymocyte globulin; hCMV, human cytomegalovirus.

**Table S7. Antibody information.**

| Manufacturer | Name | Format | Clone | Cat. |
| --- | --- | --- | --- | --- |
| Biolegend | CD3 | Alexa-Fluor®700 | SK7 | 344822 |
| Biolegend | CD4 | PerCP/Cyanine5.5 | A161A1 | 357414 |
| Biolegend | CD4 | Alexa-Fluor®700 | A161A1 | 357418 |
| BD Horizon | CD8 | V500 | RPA-T8 | 560774 |
| BD Horizon | CD8 | APC-R700 | RPA-T8 | 565165 |
| BD Pharmingen | CD45RA | PE-Cy^TM^7 | HI100 | 560675 |
| Biolegend | CD45RA | APC/Fire^TM^750 | HI100 | 304151 |
| BD Horizon | CCR7 | V450 | 150503 | 560863 |
| Biolegend | CCR7 | Brilliant Violet 421™ | G043H7 | 353208 |
| Biolegend | CD127 | PE | A019D5 | 351304 |
| Biolegend | CD25 | APC | BC96 | 302610 |
| Biolegend | HLA-DR | Pacific Blue^TM^ | L243 | 307633 |
| Biolegend | Ki-67 | APC | Ki-67 | 350514 |
| Biolegend | IFN-γ | PE/Cyanine7 | 4S.B3 | 502528 |
| Biolegend | CD31 | PE/Dazzle^TM^594 | WM59 | 303130 |
| Biolegend | PD-1 | Brilliant Violet 750™ | EH12.2H7 | 329966 |
| Biolegend | HLA-DQ | FITC | [HLADQ1](https://www.biolegend.com/en-us/search-results?Clone=HLADQ1) | 318104 |
| Biolegend | TIGIT | Brilliant Violet 605™ | A15153G | 372712 |
| Biolegend | CD27 | APC/Fire™ 810 | [O323](https://www.biolegend.com/en-us/search-results?Clone=O323) | 302864 |
| BD Pharmingen | Annexin V | APC | - | 550474 |
| BD Pharmingen | 7-AAD | PerCP | - | 559925 |
| CST | CD4 | - | D7D2Z | 25229 |
| CST | CD8a | - | D4W2Z | 98941 |
| Abcam | GFP | - | EPR14104 | ab183734 |
| Panovue | DAPI | - | - | 10012100500 |
